# Biomechanical and Microstructural Properties of Subchondral Bone From Three Metacarpophalangeal Joint Sites in Thoroughbred Racehorses

**DOI:** 10.3389/fvets.2022.923356

**Published:** 2022-06-28

**Authors:** Duncan J. Pearce, Peta L. Hitchens, Fatemeh Malekipour, Babatunde Ayodele, Peter Vee Sin Lee, R. Chris Whitton

**Affiliations:** ^1^Faculty of Veterinary and Agricultural Sciences, The University of Melbourne, Werribee, VIC, Australia; ^2^Department of Biomedical Engineering, The University of Melbourne, Parkville, VIC, Australia

**Keywords:** subchondral bone, metacarpus, equine, stiffness, hysteresis, microdamage

## Abstract

Fatigue-induced subchondral bone (SCB) injury is common in racehorses. Understanding how subchondral microstructure and microdamage influence mechanical properties is important for developing injury prevention strategies. Mechanical properties of the disto-palmar third metacarpal condyle (MCIII) correlate poorly with microstructure, and it is unknown whether the properties of other sites within the metacarpophalangeal (fetlock) joint are similarly complex. We aimed to investigate the mechanical and structural properties of equine SCB from specimens with minimal evidence of macroscopic disease. Three sites within the metacarpophalangeal joint were examined: the disto-palmar MCIII, disto-dorsal MCIII, and proximal sesamoid bone. Two regions of interest within the SCB were compared, a 2 mm superficial and an underlying 2 mm deep layer. Cartilage-bone specimens underwent micro-computed tomography, then cyclic compression for 100 cycles at 2 Hz. Disto-dorsal MCIII specimens were loaded to 30 MPa (*n* = 10), while disto-palmar MCIII (*n* = 10) and proximal sesamoid (*n* = 10) specimens were loaded to 40 MPa. Digital image correlation determined local strains. Specimens were stained with lead-uranyl acetate for volumetric microdamage quantification. The dorsal MCIII SCB had lower bone volume fraction (BVTV), bone mineral density (BMD), and stiffness compared to the palmar MCIII and sesamoid bone (*p* < 0.05). Superficial SCB had higher BVTV and lower BMD than deeper SCB (*p* < 0.05), except at the palmar MCIII site where there was no difference in BVTV between depths (*p* = 0.419). At all sites, the deep bone was stiffer (*p* < 0.001), although the superficial to deep gradient was smaller in the dorsal MCIII. Hysteresis (energy loss) was greater superficially in palmar MCIII and sesamoid (*p* < 0.001), but not dorsal MCIII specimens (*p* = 0.118). The stiffness increased with cyclic loading in total cartilage-bone specimens (*p* < 0.001), but not in superficial and deep layers of the bone, whereas hysteresis decreased with the cycle for all sites and layers (*p* < 0.001). Superficial equine SCB is uniformly less stiff than deeper bone despite non-uniform differences in bone density and damage levels. The more compliant superficial layer has an important role in energy dissipation, but whether this is a specific adaptation or a result of microdamage accumulation is not clear.

## Introduction

The metacarpophalangeal joint is the most common site of severe injury in Thoroughbred racehorses worldwide (1–6). Large peak contact forces generate microdamage in calcified cartilage that propagates into subchondral bone (SCB) (7–10). Within the joint, fatigue-related SCB microdamage is most common and severe in the disto-palmar third metacarpal condyle (MCIII), a site that articulates with the proximal sesamoid bone when the joint is under extension during midstance (11, 12). Microdamage is less severe in proximal sesamoid SCB (13), and rare in the disto-dorsal MCIII (14, 15).

Due to the high prevalence of palmar MCIII injury in the metacarpophalangeal joints of Thoroughbred racehorses, the microstructural and mechanical properties of SCB in this region have recently been investigated. At this site, the deep SCB has a higher bone mineral density (BMD), is stiffer, and dissipates less energy (hysteresis) than the superficial layer under loading (16). Except for a negative association between BMD and energy loss in the superficial SCB, correlations between microstructural and mechanical properties are poor (16). In contrast, microdamage is a good predictor of mechanical properties: stiffness decreases and energy loss increases with increasing microdamage in the palmar MCIII (17). Because the palmar aspect of the MCIII in racehorses has a high burden of microdamage it is possible that microdamage is primarily responsible for the poor correlation between microstructure and mechanical properties at this site. Knowledge of the mechanical behavior of other sites within the metacarpophalangeal joint that tend to have lower levels of microdamage would improve our understanding of this relationship.

With cyclic loading *ex vivo*, palmar MCIII SCB specimens stiffen progressively. A rapid, then more gradual increase to maximum stiffness is usually observed, likely due to the accumulation of residual strain with each subsequent cycle (18–21). Maximal stiffness occurs at a median of 24% (range 3–42%) of fatigue life, and it is maximal, not initial–stiffness, that is associated with fatigue life (20).

To further investigate the relationship between subchondral microstructure, microdamage, and mechanics within the metacarpophalangeal joint of Thoroughbred racehorses, we aimed to (1) identify differences in the microstructural and biomechanical properties of SCB between the palmar MCIII, dorsal MCIII, and proximal sesamoid bone in specimens with the minimal macroscopic disease, (2) investigate whether associations exist between microstructure and biomechanics, and (3) determine how mechanical properties change with cyclic loading. Specimens with low microdamage burdens were studied to minimize the confounding effects of damage on mechanics and help clarify whether the behavior of SCB previously observed at the palmar site is independent of damage. We hypothesized that (1) bone volume fraction (BVTV), BMD, and stiffness in the palmar MCIII and proximal sesamoid SCB would be similar, but different to the dorsal MCIII; (2) that there would be less of a difference between superficial and deep SCB stiffness in the dorsal MCIII compared to the other sites; and (3) that stiffness would increase and accordingly hysteresis would decrease with cyclic loading at all sites.

## Materials and Methods

### Specimen Preparation

Cartilage-bone specimens were collected from Thoroughbred racehorses that died or were euthanized on racetracks in Victoria, Australia (*n* = 10; Supplementary Table 1.1). At the time of post mortem, a random number generator was used to assign either the left or right forelimbs to frozen storage. From these frozen specimens and to obtain a relatively uniform sample without severe microdamage, the lateral condyle of the first 10 limbs that met eligibility criteria for this study were selected; horses aged 3–4 years and grade 1 or less palmar osteochondral disease grade (POD; Supplementary Table 1.2) (11, 12, 22). Three left and seven right forelimbs were used. The average age of horses at the time of death was 3.66 (±0.42) years old. Four horses were female and six male, of which three were gelded and three were entire. The causes of death were euthanasia due to catastrophic musculoskeletal injury (*n* = 5) and sudden death or pulmonary edema (*n* = 5).

**Figure 1:**
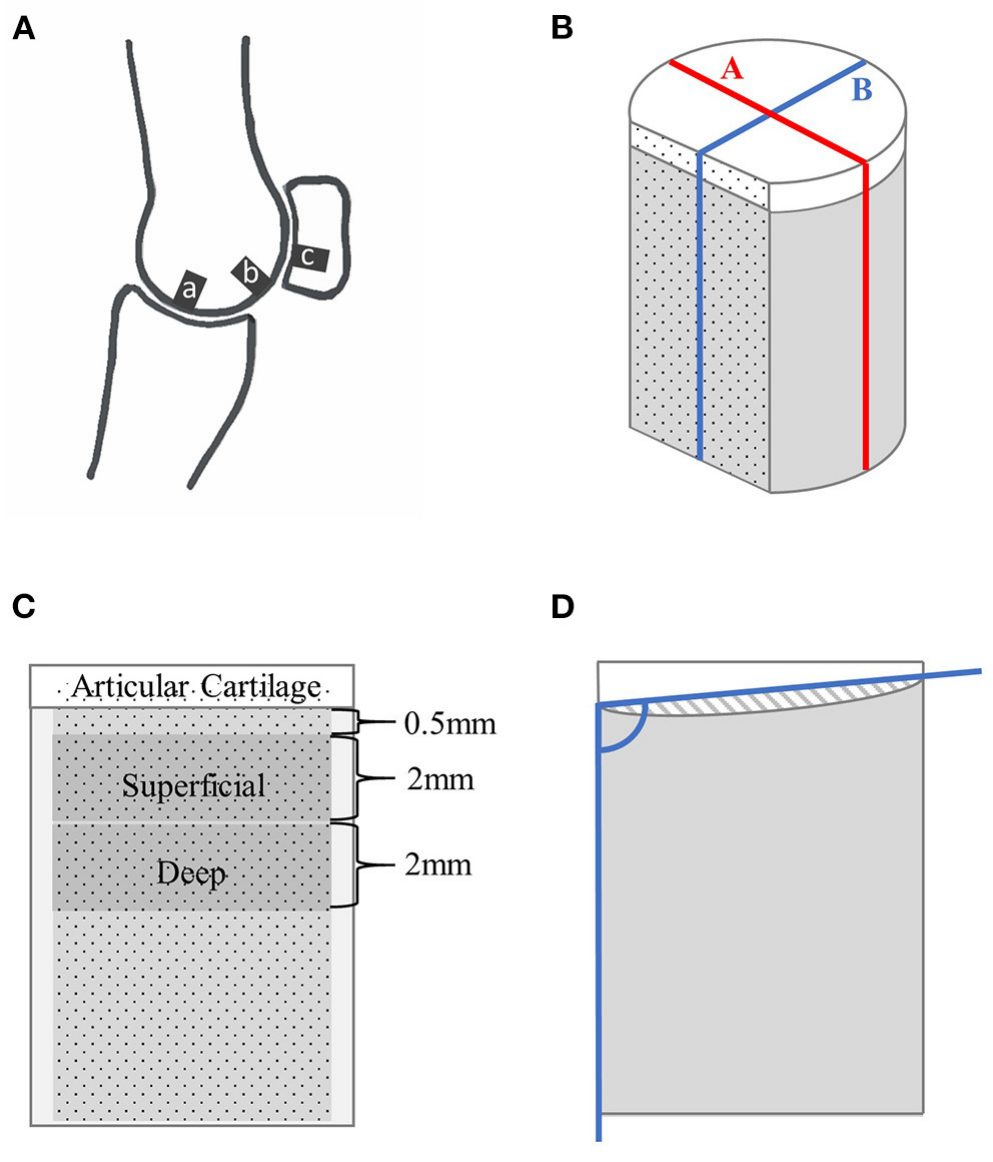
Schematic drawings showing the locations of specimen collection within a fetlock joint **(A)** and of a cartilage-bone specimen where nonmineralized cartilage is represented in white, and mineralized tissue in gray **(B–D)**. **(A)** Parasagittal view through the lateral condyle of a fetlock joint, where **‘a’** is the disto-dorsal MCIII, **‘b’** the disto-palmar MCIII, and **‘c’** the proximal sesamoid specimen collection sites. **(B)** The cylindrical specimen with a flat cut face, and lines ‘A’ and ‘B’. **(C)** View of the stained face showing the 2 mm thick superficial and deep layers. **(D)** Sagittal view along line ‘B’ showing the angle used to calculate mineralized tissue surface angle, and the cross-sectional shaded area of hyaline cartilage used to measure surface evenness.

Specimens were collected from three sites: the disto-dorsal aspect of the lateral MCIII, the disto-palmar aspect of the lateral MCIII condyle, and the articular surface of the lateral proximal sesamoid bone, for a total of n = 30 specimens (Figure 1A). A diamond-coated core drill (#102075, Starlite Industries Inc, Rosemont, Pennsylvania, USA) was used to cut approximately 6.7 mm diameter cylindrical specimens from the palmar MCIII, dorsal MCIII, and proximal sesamoid bones. The specific sites chosen for the collection were approximately 5 mm palmar to the transverse ridge and 1/3 of the distance from the condylar groove to the abaxial margin; approximately, 17 mm dorsal to the palmar collection site and midway between the condylar groove and abaxial margin; and the lateral articular concavity within the proximal sesamoid bone. Specimens were stored frozen in Compound Sodium Lactate solution [(Hartmann's) Fresenius Kabi Deutschland, Friedberg, Germany].

Prior to imaging, specimens were cut to an approximate length of 10 mm using a slow speed diamond wavering blade saw (IsoMet, Buehler, Lake Bluff, Illinois, USA) with saline lavage, and a vertical section approximately 1.0 mm thick was removed from the cartilage-bone to create a flat surface perpendicular to the articular surface as previously described (Figure 1B) (16). This flat plane was later airbrushed with a black random speckle pattern (Ophir Airbrush, Mission Viejo, CA, USA; OP-180KT) to facilitate digital image correlation.

### MicroCT

Cartilage-bone specimens were analyzed in a micro-computed tomography (microCT) scanner (μCT50, Scanco Medical AG, Brüttisellen, Switzerland) at a resolution of 4.0 μm using 70 kVp tube voltage, 200 μA tube current, a 0.5 mm aluminum filter, and 600 ms integration time. Phantoms of 0.25 and 0.75 g/ccm calcium hydroxyapatite were used to calibrate greyscale units to equivalent bone mineral density (CTan 1.13.15.1+; Bruker microCT, Billerica, Massachusetts, USA). Specimens were re-frozen after scanning.

Bone volume fraction (bone volume/total volume, the inverse of porosity) and bone mineral density [in mg hydroxyapatite(HA)/ccm] were determined for mineralized tissue of the total cartilage-bone specimen and two regions of interest: a superficial 2.0 mm section beginning at 0.5 mm below the mineralized–non-mineralized tissue interface, and a deep 2.0 mm section immediately below the superficial section (Figure 1C). These two regions of interest were chosen so that density and stiffness gradients could be examined and to facilitate comparison with the literature (16). Regions of interest were segmented 1.0 mm from the cut edges to exclude pores filled with debris. Scanco Medical μCT software was used, with an upper threshold of 1,000 and a lower threshold refined individually for each specimen in the superficial and deep layer, with previews visually assessed and compared to greyscale images; a range between 233 and 282 was used. All pixels within the greyscale regions were assigned as mineralized tissue, whereas all pixels below the lower end of the range were set as background. Gauss sigma and support were fixed for all morphometry calculations at 1 and 2, respectively. These were chosen to closely match the visual smoothness of the microCT images.

MicroCT slices were assessed for microdamage in the transverse, frontal, and sagittal planes using image analysis software [Fiji (23)]. A microfracture was recorded if a localized linear lucency, or linear hypermineralization, was observed in two planes and extended across multiple slices (9). Microfractures were graded semi-quantitatively as 0 if absent, and 1 if one or more were present. Resorption was graded as 0 if absent, and 1 if the specimen had subjectively greater regional lucency than expected. For both microfracture and resorption grades, overall scores were given to each specimen and applied to all regions of interest for the purposes of statistical analysis. Average cartilage thickness was calculated along two lines: line A–parallel to the flat plane, and at the widest point of the specimen; and line B–perpendicular to the flat plane, and at the widest point (Figure 1B). Surface evenness was assessed in two ways, first by measuring mineralized tissue surface angle relative to a side of the specimen across lines A and B; and second, mineralized tissue surface convexity or concavity was assessed by calculating the area between (above or below) the mineralized tissue surface and the lines used to measure surface angle (Figure 1D).

### Mechanical Testing

Cartilage-bone specimens were tested under unconfined cyclic compression. They were mounted with a thin layer of cyanoacrylate glue (Henkel Loctite Super Glue, 161942 SG3) on a custom fitting comprised of a stainless-steel base and a clear plastic chamber. This was attached to an ElectroForce® 3500 load cell (TA Instruments, New Castle, Delaware, USA), and specimens were then submerged in physiologic saline [Compound Sodium Lactate solution (Hartmann's), Fresenius Kabi Deutschland, Friedberg, Germany]. A small preload of 20 N (~0.65 MPa) for 5 min was applied to ensure good contact between the platen and specimen. Testing was performed with sinusoidal waveforms of 1 to 30 MPa for dorsal MCIII specimens and 1 to 40 MPa for palmar MCIII and proximal sesamoid specimens. This load is approximately equivalent to that experienced by the joint when a horse is trotting (24). It was chosen to minimize the accumulation of microdamage during *ex vivo* testing and is comparable to a previous study (16). One hundred cycles were performed at a frequency of 2 Hz. One hundred compressive cycles were chosen to provide sufficient cycles for initial rapid stiffening to occur, followed by a more gradual increase without causing undue *ex vivo* damage (25). Displacement and load data points were collected at a frequency of 500 Hz for the duration of the test.

### Image Capture and Digital Image Correlation

During testing, the flat painted cartilage-bone surface was imaged with a digital high-speed camera, as previously described (17). Images were captured at 500 frames per second. Digital image correlation analysis (VIC-2D 6, Correlated Solutions, Inc., Irmo, South Caroline, USA) is a non-contact technique used to measure in-plane displacements using optical cameras and was performed on cycles 1, 2, 3, 5, and 9. These cycles were selected to evaluate early loading in detail. Camera memory, software, and time constraints precluded analysis of all cycles. Displacement data was processed in MATLAB (The MathWorks, Inc. Version 2017b) to calculate strain in the superficial and deep regions of interest, by dividing the relative displacement of the upper and lower bounds by the distance between them (2 mm). Stress and strain data were matched by comparing peak actuator displacement (and correlating stress) from the ElectroForce® 3500 output with peak actuator displacement in VIC-2D.

### Stiffness, Hysteresis, and Strain

Total cartilage-bone specimen stiffness (Young's modulus) was calculated from displacement and load data in Microsoft Excel (Version 1803) for cycles 1, 2, 3, 5, 9, 19, 29, 39, 49, 59, 69, 79, 89, and 99. The slope of the middle 50% of a loading curve was specified as stiffness, using least squares fit. Superficial and deep specimen stiffness were calculated for cycles 1, 2, 3, 5, and 9 in MATLAB. Normalized hysteresis (fraction of energy loss) was calculated for cycles 1, 2, 3, 5, and 9 in superficial and deep bone sections, and cycles 1, 2, 3, 5, 9, 19, 29, 39, 49, 59, 69, 79, 89, and 99 for total cartilage-bone sections using the trapezoid method in Microsoft Excel. Engineering strain was calculated as the change in length during loading divided by length prior to loading. Timepoints for analysis were chosen to evaluate early loading in detail. Time constraints precluded analysis of all cycles, and camera memory and software constraints limited the number of superficial and deep cycles that could be analyzed.

### Lead-Uranyl Acetate Staining and Repeat MicroCT

Staining specimens with lead-uranyl acetate was performed to allow detection of diffuse microdamage by binding to exposed phosphate on the bone surface (26). Cartilage-bone plugs were thawed and incubated in 8% uranyl acetate in 70% acetone for 7 days, followed by a 70% acetone wash. Specimens were subsequently incubated in 20% lead acetate and 70% acetone under vacuum for 12 h, then placed on a shaker for 7 days. Following this incubation, specimens were washed twice per day in 70% acetone with 30-min sonication for a total of 14 days. Lastly, an incubation in 1% ammonium sulfide in acetone for 1 day was followed by a final wash in 70% acetone. Specimens were stored in acetone until imaging.

All specimens were then microCT scanned again. Specimen segmentation (superficial and deep, but not total bone) was performed at a similar lower threshold level based on the visual assessment as used pre-label and bone volume reported. This threshold level included labeled and unlabelled bone. Segmentation was then performed at a high threshold (550) to select only labeled bone. The region of interest was an area of 1.0 mm from the cut edge of specimens to exclude label accumulation on cut surfaces. Morphometric evaluations were also performed to calculate the bone surface area (BSA) mm^2^; this value was available for all superficial and deep layers in specimens except the deep layer in one dorsal MCIII specimen. The fraction of labeled (or damaged) bone was calculated as:


(1) $$\begin{array}{l} \text{Damage bone volume fraction }=\;\frac{\text{damage bone volume }\left(\text{m}{\text{m}}^{3}\right)}{\text{bone volume }\left(\text{m}{\text{m}}^{3}\right)} \end{array}$$

In highly porous specimens, labels tended to line open pores. Therefore, an adjustment was performed by dividing the fraction of labeled bone by the bone surface area:


(2) $$\begin{array}{l} \text{Adjusted damaged bone volume }\left(\text{m}{\text{m}}^{-2}\right)=\\ \frac{\text{damaged bone volume fraction}}{\text{bone surface area }\left(\text{m}{\text{m}}^{2}\right)} \end{array}$$

### Statistical Analysis

The sample size required to identify a difference in mean parameters between the three groups was assessed using G^*^Power 3.1.9.4, with power set at 80% and a significance level of 5% (27). Using published values for the main variables of interest–BVTV, BMD, and stiffness–five samples per group would be required to detect a 10% group mean difference (15, 16, 28, 29).

Generalized linear mixed models were generated to assess (1) bone microstructure and (2) bone mechanics. Univariable models were initially generated for each site (dorsal MCIII, palmar MCIII, proximal sesamoid) and layer (superficial, deep, total cartilage-bone). In the bone microstructure models, dependent continuous variables included BVTV, BMD, damaged bone volume fraction, and adjusted damaged bone volume. In the bone mechanics models, stiffness and normalized hysteresis were considered dependent continuous variables. Cycle number was initially assessed as either a continuous and categorical variable, then due to its non-linear relationship with the dependent variable, included as a fixed-effect categorical variable for cycles 1, 2, 3, 5, and 9 in the superficial and deep SCB models, and cycles 1, 2, 3, 5, 9, 19, 29, 39, 49, 59, 69, 79, 89, and 99 in the total cartilage-bone specimen models.

Both sets of models were adjusted for horse-level random effects to account for multiple measures within horses at different sites, layers, and repeated across cycles. Continuous independent variables assessed were as follows: BVTV, BMD, damaged bone volume fraction, adjusted damaged bone volume, age, cartilage thickness, angle A, angle B, evenness area A, and evenness area B. Categorical variables assessed were as follows: site (dorsal MCIII, palmar MCIII, proximal sesamoid), layer (superficial, deep), horse sex, limb side, grade of POD, grade of microfracture, fracture as the cause of death, and grade of resorption. Two-way interactions were assessed between biologically plausible main effects. Two-way interactions between site and multiple other independent variables were identified in models for stiffness and hysteresis in the superficial and deep SCB, therefore models were stratified by site. Thus, there were 100 observations in superficial and deep SCB models (10 horses, one site, two layers, and five cycles) and 420 observations in the total cartilage-bone models (10 horses, three sites, one layer, and 14 cycles). Variables that were *p* < 0.20 in univariable modeling were fitted into a multivariable model and retained if *p* < 0.05 using the backward stepwise method. Models were generated with and without two specimens (an outlier where total specimen BVTV = 0.61 and a specimen with microfracture) and were not found to significantly influence the final outcome. Correlated independent variables identified using pairwise Pearson's correlation (rho > 0.60) were assessed independently in the models, with the best fitting variable according to lowest AIC/BIC retained.

Univariable statistical analysis on microstructural outcome variables was performed using SPSS® Statistics Version 26 (IBM Corporation, Chicago, Illinois, USA). Univariable and multivariable analysis on mechanical outcome variables was performed using Stata/SE 15.1 (StataCorp. 2015. Stata Statistical Software: Release 15. College Station, Texas, USA: StataCorp LP).

## Results

### Descriptive Statistics

**Figure 2:**
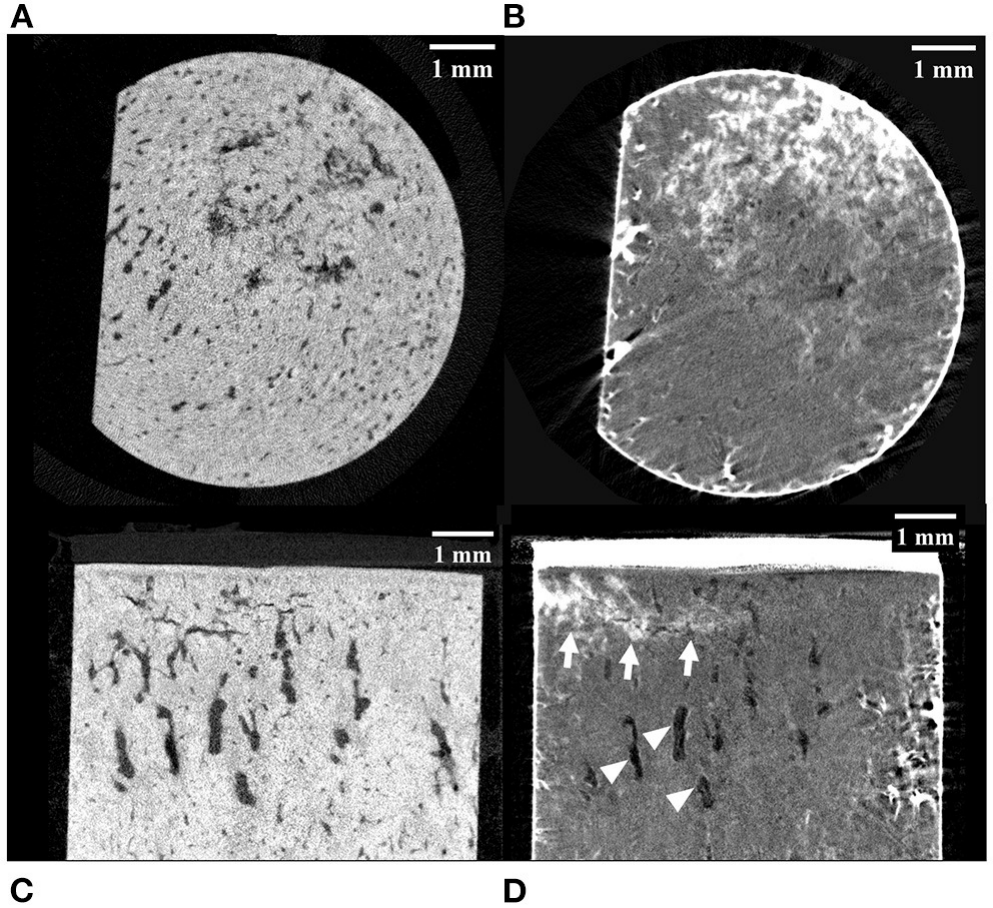
MicroCT images of a palmar MCIII cartilage-bone specimen from a Thoroughbred racehorse with microfractures before **(A,C)** and after **(B,D)** staining the bone with lead-uranyl acetate. Image **(A)** and **(B)** are short axis images taken approximately 1 mm below the mineralized to non-mineralized cartilage interface. Images **(C)** and **(D)** are long axis images of the superficial half of the specimen. Image **(A)** was taken at an approximately equivalent slice as image **(B)**; likewise, with images **(C)** and **(D)**. The staining in images **(B)** and **(D)** confirms uptake in this specimen with known microdamage (arrows). Resorptive lesions are present (arrowheads).

**Figure 3:**
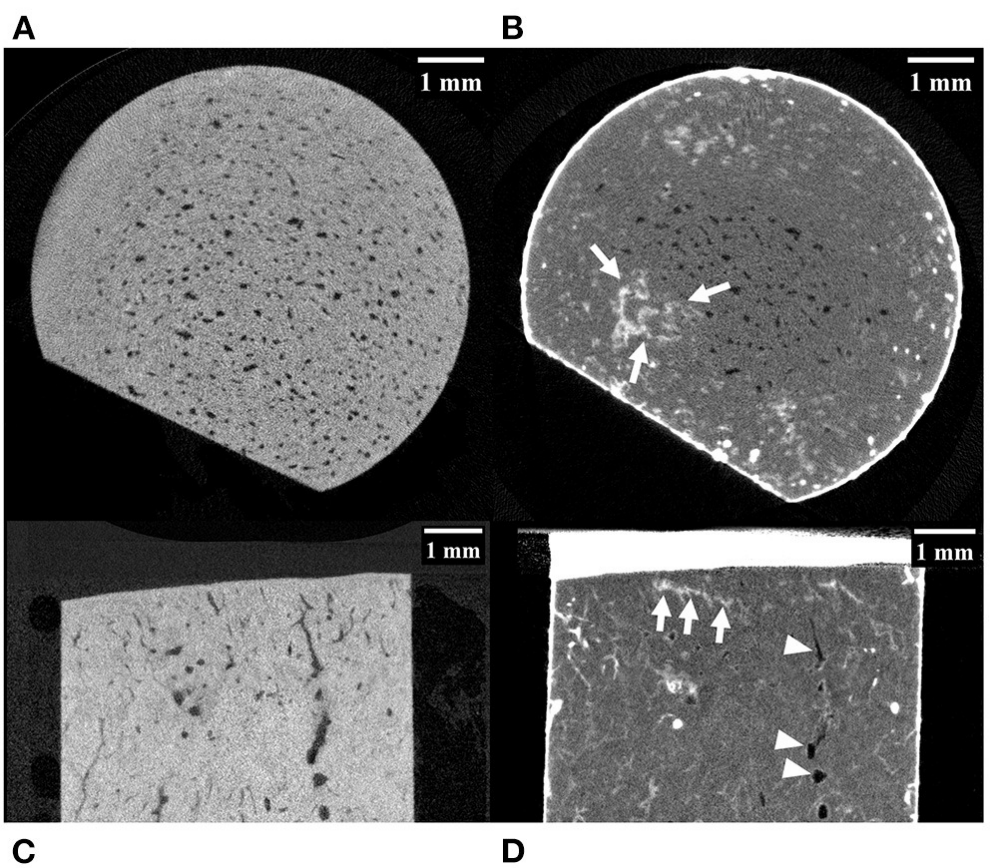
MicroCT images of a palmar MCIII cartilage-bone specimen from a Thoroughbred racehorse. **(A,C)** are unlabelled images without evidence of microfractures, while **(B,D)** are labeled images taken at approximately equivalent slices after staining the bone with lead-uranyl acetate showing small areas of label uptake near the articular surface consistent with microdamage (highlighted by white arrows). Image **(A,B)** are short axis images taken approximately 0.5 mm below the mineralized to non-mineralized cartilage interface. Images **(C,D)** are long axis images of the superficial half of the specimen. Resorptive lesions are present (arrowheads).

Of the 10 horses, specimens were collected from seven with no gross evidence of joint surface injury (POD) and three with joint surface discoloration (grade 1 POD). The articular cartilage of all specimens was structurally intact. Evidence of two or more microfractures was observed in only one palmar specimen on microCT (Figures 2A,C). Processing specimens with lead uranyl acetate enabled the identification of more diffuse and extensive microdamage in one specimen with microfractures visible on plain microCT (Figure 2). It also enabled the identification of a small area of superficial, linear, and oblique label uptake consistent with microdamage in a palmar MCIII specimen without microfractures seen on plain microCT (Figure 3). This pattern was not seen in sesamoid or dorsal MCIII specimens.

Specimen SCB height was a mean of 10.12 ± 0.37 mm, specimen diameter a mean of 6.66 ± 0.05 mm, and the width of the flat cut plane was a mean of 4.95 ± 0.19 mm. The mean non-mineralized cartilage thickness was 0.56 ± 0.09 mm. Mineralized tissue surface angle along the line ‘A’ (parallel to the cut face, and at the widest point of the specimen) was a mean of 89.84 ± 1.61 degrees and along the line ‘B’ (perpendicular to the cut face, and at the widest point) was a mean of 89.79 ± 2.41 degrees relative to the sides of the specimens. Mineralized tissue surface concavity and convexity along the line ‘A’ and ‘B’ were 0.52 ± 0.95 mm^2^ and−0.04 ± 0.41 mm^2^, respectively, where a negative value indicated concavity and a positive value convexity.

Descriptive statistics for BVTV, BMD, damaged bone volume fraction, and adjusted damaged bone volume are presented in Table 1, and descriptive statistics for first cycle apparent strain (%), stiffness (MPa), and normalized hysteresis (fraction) are presented in Table 2.

**Table 1:** Descriptive statistics for bone volume fraction [bone volume mm^3^ / total volume mm^3^], bone mineral density [mg HA/ccm], damaged bone volume fraction [damaged bone volume mm^3^ / bone volume mm^3^], and adjusted damaged bone volume fraction [damaged bone volume fraction/bone surface area mm^2^] by the site (dorsal MCIII, palmar MCIII, and proximal sesamoid) and layer (superficial 2 mm, deeper 2 mm, and total ~10 mm thick specimen) from samples taken from the metacarpophalangeal joints of Thoroughbred racehorses at post-mortem (*n* = 10, except for the damaged bone volume and adjusted damaged bone volume at the dorsal deep site where *n* = 9).

		**Bone volume fraction**			**Bone mineral density (mg HA/ccm)**			**Damaged bone volume fraction**			**Adjusted damaged bone volume (mm** ^**−2**^ **)**		
**Site**	**Layer**	**Mean**	**sd**	**CoV**	**Mean**	**sd**	**CoV**	**Mean**	**sd**	**CoV**	**Mean**	**sd**	**CoV**
**Dorsal**													
	Deep	0.68	0.08	12.2%	898.71	27.24	3.0%	0.01	0.006	42.0%	0.0001	0.00003	42.8%
	Superficial	0.80	0.10	12.2%	856.21	25.02	2.9%	0.03	0.01	50.5%	0.0002	0.0002	74.3%
	Total	0.71	0.08	10.7%	891.19	30.13	3.4%	-	-	-	-	-	-
**Palmar**													
	Deep	0.91	0.10	11.2%	921.86	25.40	2.8%	0.02	0.01	61.3%	0.0005	0.0005	99.9%
	Superficial	0.94	0.09	9.2%	882.34	28.44	3.2%	0.02	0.01	67.2%	0.0003	0.0002	75.8%
	Total	0.87	0.10	11.4%	913.29	22.66	2.5%	-	-	-	-	-	-
**Sesamoid**													
	Deep	0.85	0.05	6.3%	945.54	14.00	1.5%	0.03	0.01	34.6%	0.0004	0.0002	59.2%
	Superficial	0.94	0.04	4.0%	906.62	16.24	1.8%	0.04	0.01	37.2%	0.0007	0.0004	53.1%
	Total	0.83	0.05	5.7%	928.29	12.60	1.4%	-	-	-	-	-	-

*sd, Standard Deviation; CoV, Coefficient of Variation*.

**Table 2:** Descriptive statistics of first cycle apparent strain (%), stiffness (MPa), and normalized hysteresis (fraction) by the site (dorsal MCIII, palmar MCIII, and proximal sesamoid) and layer (superficial 2 mm, deeper 2 mm, and total ~10 mm thick specimen) from cartilage-bone samples taken from the metacarpophalangeal joints of Thoroughbred racehorses at post-mortem (*n* = 10).

		**Apparent strain (%)**			**Stiffness (MPa)**			**Normalized hysteresis (fraction)**		
**Site**	**Layer**	**Mean**	**sd**	**CoV**	**Mean**	**sd**	**CoV**	**Mean**	**sd**	**CoV**
**Dorsal**										
	Deep	0.53	0.13	24.2%	6063.00	1310.62	21.6%	0.12	0.08	69.8%
	Superficial	0.78	0.19	24.2%	4345.10	926.65	21.3%	0.29	0.08	27.8%
	Total	1.47	0.23	15.8%	2170.70	334.71	15.4%	0.26	0.05	20.4%
**Palmar**										
	Deep	0.49	0.12	25.3%	8643.80	2137.19	24.7%	0.18	0.05	28.3%
	Superficial	0.92	0.30	32.9%	5059.00	1392.97	27.5%	0.36	0.10	28.8%
	Total	1.44	0.31	21.6%	3007.20	523.80	17.4%	0.29	0.07	23.3%
**Sesamoid**										
	Deep	0.43	0.08	19.8%	9288.50	1689.15	18.2%	0.12	0.06	47.1%
	Superficial	0.93	0.30	32.0%	4890.30	1286.01	26.3%	0.38	0.11	29.0%
	Total	1.62	0.32	19.9%	2721.05	523.24	19.2%	0.34	0.07	20.4%

*sd, Standard Deviation; CoV, Coefficient of Variation*.

In the proximal sesamoid specimens, independent variables significant and highly correlated (rho > 0.60) with each other included BVTV and adjusted damaged bone volume (rho = 0.681). In the dorsal MCIII, they included age and mineralized tissue surface concavity and convexity along the line ‘B’ (rho = 0.682), age and adjusted damaged bone volume (rho = 0.603), cartilage thickness, and mineralized tissue surface angle along the line ‘B’ (rho = 0.654), mineralized tissue surface concavity and convexity along the line ‘A’ and ‘B’ (rho = −0.679), and mineralized tissue surface concavity and convexity along the line ‘B’ and damaged bone volume fraction (rho = 0.781). In the palmar MCIII, age and mineralized tissue surface concavity and convexity along the line ‘A’ were correlated (rho = − 0.772).

### Bone Microstructural Properties of Fetlock Joint Sites

**Figure 4:**
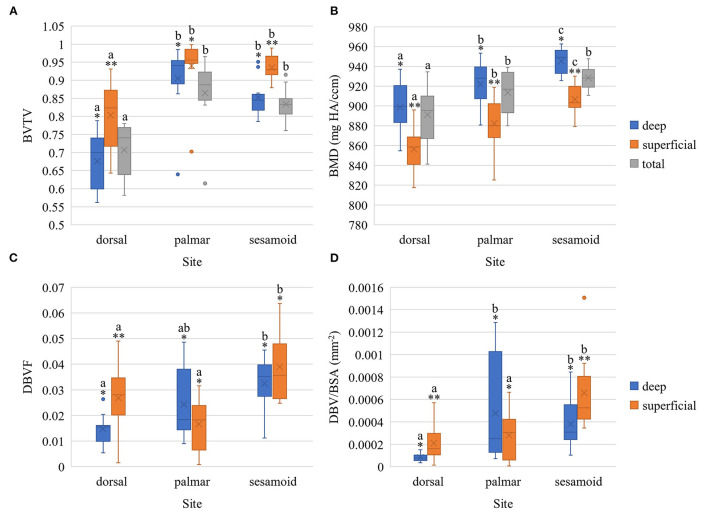
Boxplots of bone volume fraction **(A)**, bone mineral density **(B)**, damaged bone volume fraction **(C)**, and adjusted damaged bone volume fraction **(D)** within the metacarpophalangeal joint of Thoroughbred racehorses [*n* = 10 except for **(C)** and **(D)** where *n* = 9 in the deep dorsal site] stratified by site (palmar MCIII, dorsal MCIII, proximal sesamoid) and by layer (superficial 2 mm, deeper 2 mm, or total ~10 mm thick specimen). BVTV, bone volume fraction; BMD, bone mineral density in mg HA/ccm; DBVF, damaged bone volume fraction; DBV/BSA, adjusted damaged bone volume fraction in mm^−2^; X represents the mean, the horizontal midpoint of the box the median, the lower end of the box the first quartile, the upper end of the box the third quartile, and the “whiskers” extend from the ends of the box to the maximum and minimum values. The dots signify outlier values. ^*,**^ Within each site, layers with different asterisk annotations are significantly different (*P* < 0.05). Total specimen BVTV and BMD was not compared to superficial and deep as it comprises both. ^a,b,c^ Within each layer, sites with different alphabetical annotations are significantly different (*P* < 0.05).

Bone volume fraction was lower in the dorsal MCIII compared to the palmar MCIII and proximal sesamoid sites at all layers of bone: total, superficial, and deep (*p* < 0.05; Figure 4; Supplementary Table 2.1). The superficial layer had higher BVTV than the deep layer for dorsal (*p* = 0.001) and sesamoid (*p* = 0.006) sites, but not the palmar site (*p* = 0.419; Figure 4; Supplementary Table 2.2).

Bone mineral density was greatest in the proximal sesamoid bone, followed by palmar MCIII, and the lowest in the dorsal MCIII (Figure 4). These differences were significant at all layers (*p* < 0.05; Supplementary Table 2.3) except for between palmar MCIII and proximal sesamoid bone for total bone specimens (*p* = 0.156). Bone mineral density in the deep SCB was higher than in the superficial SCB within all sites: sesamoid (*p* < 0.001), palmar (*p* = 0.004), and dorsal (*p* = 0.002; Figure 4; Supplementary Table 2.4).

Figure 4 and Supplementary Tables 2.5, 2.6 present differences in damaged bone volume across sites and layers. Adjusted damaged bone volume was higher in the superficial and deep sesamoid bone compared to the dorsal MCIII (*p* < 0.001 and *p* = 0.046 respectively; Supplementary Table 2.7). Superficially, the sesamoid adjusted damaged bone volume was also higher than in the palmar MCIII bone (*p* = 0.002), while the deep SCB-adjusted damaged bone volume was higher in the palmar MCIII compared to the dorsal site (*p* = 0.010). Within sesamoid and dorsal MCIII specimens, the superficial bone had higher adjusted damaged bone volume compared to the deeper bone (*p* = 0.048 and *p* = 0.027 respectively; Supplementary Table 2.8).

### Bone Mechanical Properties of Fetlock Joint Sites

#### Associations With Stiffness

Univariable associations between total cartilage-bone specimen stiffness and study factors are presented in Supplementary Table 3.1. In multivariable modeling, cycle, BVTV, BMD, site, presence of microfracture, cartilage thickness, and mineralized tissue surface evenness were associated with total cartilage-bone specimen stiffness (Table 3). Interactions were present, whereby BVTV, BMD, cartilage thickness, and surface evenness along the line B differed by the site (dorsal MCIII, palmar MCIII, or proximal sesamoid). The stiffness increased with the cycle at all sites. The stiffness increased linearly with increasing BVTV at all sites, thus the lower overall stiffness in the dorsal MCIII site was largely explained by lower BVTV in this section of bone. There was a positive association between stiffness and BMD in the proximal sesamoid bone, a weak positive association in the palmar MCIII bone, and a weak negative association in dorsal MCIII bone. The whole specimen with microfracture was significantly stiffer than specimens without. Overall, non-mineralized cartilage thickness was negatively associated with stiffness, and this association was weakest in sesamoid specimens. Mineralized tissue surface evenness along the line ‘B’ (where negative values signify concavity and positive convexity) at dorsal and sesamoid sites was positively associated with stiffness (and stiffness greater at any given evenness along the line ‘B’ value in the sesamoid), whereas at the palmar location the association was negative.

**Table 3:** Multivariable mixed effects linear model estimated regression coefficients (Coef.), their 95% confidence intervals, and alpha level (*p*-value) of factors associated with total ~10 mm thick cartilage-bone specimen stiffness (*n* = 420) in three sites (dorsal MCIII, palmar MCIII, and proximal sesamoid) within the metacarpophalangeal joint of *n* = 10 Thoroughbred racehorses.

**Variable**	**Coef**.	**95% Confidence interval**		***p-*Value**
		**Lower bound**	**Upper bound**	
BVTV	10551.15	9648.55	11453.74	<0.001
**Site**				
Sesamoid	Reference			
Dorsal	46626.01	42854.67	50397.35	<0.001
Palmar	37893.56	34588.18	41198.94	<0.001
**Site # BVTV interaction**				
Sesamoid # BVTV	Reference			
Dorsal # BVTV	−1831.49	−2619.23	−1043.75	<0.001
Palmar # BVTV	−6664.29	−7349.06	−5979.52	<0.001
BMD	39.48	35.39	43.57	<0.001
**Site # BMD interaction**				
Sesamoid # BMD	Reference			
Dorsal # BMD	−48.48	−52.13	−44.84	<0.001
Palmar # BMD	−34.32	−37.46	−31.18	<0.001
**Microfracture**				
0	Reference			
1	469.23	315.43	623.02	<0.001
Cartilage thickness (mm)	−981.47	−1295.77	−667.16	<0.001
**Site # Cartilage interaction**				
Sesamoid # Cartilage	Reference			
Dorsal # Cartilage	−1635.79	−2282.41	−989.17	<0.001
Palmar # Cartilage	−1769.55	−2206.14	−1332.97	<0.001
Even B	543.00	361.34	724.66	<0.001
**Site # Even B interaction**				
Sesamoid # EvenB	Reference			
Dorsal # EvenB	537.59	286.87	788.32	<0.001
Palmar # EvenB	−1550.03	−1814.25	−1285.80	<0.001
**Cycle**				
1	Reference			
2	297.79	241.74	353.85	<0.001
3	330.67	274.61	386.73	<0.001
5	368.14	312.08	424.19	<0.001
9	412.21	356.16	468.27	<0.001
19	472.53	416.48	528.59	<0.001
29	516.71	460.65	572.76	<0.001
39	550.72	494.67	606.78	<0.001
49	581.80	525.75	637.86	<0.001
59	610.76	554.70	666.82	<0.001
69	634.92	578.86	690.97	<0.001
79	659.18	603.13	715.24	<0.001
89	680.88	624.82	736.93	<0.001
99	700.76	644.70	756.81	<0.001
Constant	−41600.00	−45600.00	−37700.00	<0.001
Constant	5.870	5.444	6.330	
Constant	4.707	4.639	4.776	

**Figure 5:**
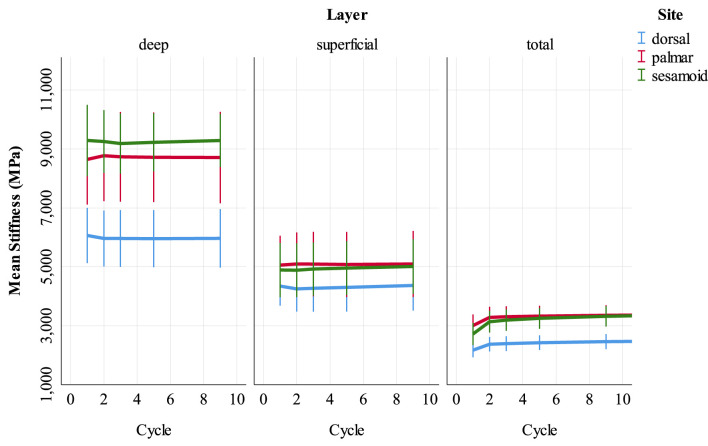
Stiffness (MPa) of cartilage-bone specimens over the first nine compressive loading cycles at three sites (dorsal MCIII, palmar MCIII and proximal sesamoid) within the metacarpophalangeal joint of *n* = 10 Thoroughbred racehorses. Layers of each specimen (superficial 2 mm, deeper 2 mm, and total ~10 mm thick cartilage-bone) are stratified into columns. Unadjusted means with 95% confidence intervals are displayed.

Univariable associations between superficial and deep SCB stiffness and study factors are presented in Supplementary Item 3 (Supplementary Tables 3.2–3.4). In multivariable analysis, stiffness was greater in deep, compared to superficial SCB at all sites (*p* < 0.001, Supplementary Tables 3.5–3.7). The magnitude of this effect was lower in the dorsal MCIII. Unlike the results for whole samples, stiffness did not differ from cycles 1 to 9 at any site (Figure 5). Higher BVTV was associated with greater stiffness in palmar MCIII specimens (*p* < 0.001, Supplementary Figure 3.1). Palmar and dorsal MCIII specimens with higher BMD were stiffer (*p* < 0.001, Supplementary Figure 3.1). In the palmar MCIII, this relationship was quadratic, whereby in specimens with higher BMD, BMD had a greater effect on stiffness. While not included in the final sesamoid model, BMD was collinear and interchangeable with BVTV, DBVF, and DBV/BSA. Associations between DBV/BSA and stiffness were not consistent between sites with a negative association in the deep layer of the dorsal MCIII (*p* < 0.001, Supplementary Figure 3.2), a predominantly positive association in the sesamoid (*p* < 0.001, Supplementary Figure 3.2) and no association in the palmar site (*p* = 0.074). In the sesamoid bone, deep, but not superficial, stiffness increased with greater cartilage thickness (*p* < 0.001), and stiffness was lower in entire males (*p* < 0.05), and in younger and older horses (*p* < 0.001).

#### Associations With Hysteresis

**Figure 6:**
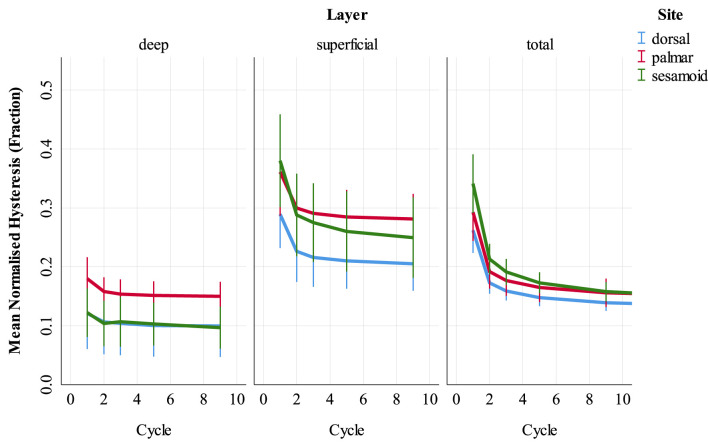
Normalized hysteresis (fraction of energy loss) of cartilage-bone specimens over the first nine compressive loading cycles at three sites (dorsal MCIII, palmar MCIII and proximal sesamoid) within the metacarpophalangeal joint of *n* = 10 Thoroughbred racehorses. Layers of each specimen (superficial 2 mm, deeper 2 mm, and total ~10 mm thick cartilage-bone) are stratified into columns. Unadjusted means with 95% confidence intervals are displayed. Note that in the deep layer, dorsal (blue) hysteresis is obscured by sesamoid (green) hysteresis.

Univariable associations between total cartilage-bone specimen hysteresis and study factors are presented in Supplementary Table 4.1. In multivariable modeling, cycle, BMD, site, cartilage thickness, and presence of microfracture were associated with total cartilage-bone specimen hysteresis (Table 4). The cycle was associated with hysteresis for all sites with a rapid initial decrease and then a more gradual decline (Figure 6). Interactions were present, whereby there was a negative association between BMD and hysteresis in the sesamoid bone but not in the other sites. A positive association between cartilage thickness and hysteresis was observed in palmar and dorsal MCIII but not proximal sesamoid specimens.

**Table 4:** Multivariable mixed effects linear model estimated regression coefficients (Coef.), their 95% confidence intervals, and alpha level (*p*-value) of factors associated with a total ~10 mm thick cartilage-bone specimen normalized hysteresis (*n* = 420) in three sites (dorsal MCIII, palmar MCIII, and proximal sesamoid) within the metacarpophalangeal joint of *n* = 10 Thoroughbred racehorses.

**Variable**	**Coef**.	**95% Confidence Interval**		***p*-Value**
		**Lower Bound**	**Upper Bound**	
BMD	−0.003	−0.003	−0.002	<0.001
**Site**				
Sesamoid	Reference			
Dorsal	−2.58	−3.11	−2.05	<0.001
Palmar	−2.40	−2.85	−1.96	<0.001
**Site # BMD interaction**				
Sesamoid#BMD	Reference			
Dorsal#BMD	0.003	0.002	0.003	<0.001
Palmar#BMD	0.002	0.002	0.003	<0.001
Cartilage thickness (mm)	−0.03	−0.08	0.02	0.261
**Site # Cartilage interaction**				
Sesamoid#Cartilage	Reference			
Dorsal#Cartilage	0.26	0.16	0.36	<0.001
Palmar#Cartilage	0.38	0.31	0.45	<0.001
**Microfracture**				
0	Reference			
1	0.03	0.02	0.05	<0.001
**Cycle**				
1	Reference			
2	−0.11	−0.12	−0.10	<0.001
3	−0.12	−0.13	−0.11	<0.001
5	−0.14	−0.15	−0.13	<0.001
9	−0.15	−0.16	−0.14	<0.001
19	−0.16	−0.17	−0.15	<0.001
29	−0.16	−0.17	−0.15	<0.001
39	−0.17	−0.18	−0.16	<0.001
49	−0.17	−0.18	−0.16	<0.001
59	−0.17	−0.18	−0.16	<0.001
69	−0.17	−0.18	−0.16	<0.001
79	−0.17	−0.18	−0.16	<0.001
89	−0.17	−0.18	−0.16	<0.001
99	−0.17	−0.18	−0.16	<0.001
Constant	2.86	2.31	3.40	<0.001
Constant	−3.97			
Constant	−3.94			

Univariable associations between superficial and deep SCB hysteresis and study factors are presented in Supplementary Item 4 (Supplementary Tables 4.2–4.4). In multivariable analysis (Supplementary Tables 4.5–4.7), hysteresis was greater in superficial, compared to deep SCB in the palmar MCIII and proximal sesamoid (*p* < 0.001) but not the dorsal MCIII (*p* = 0.118). Hysteresis decreased from cycles 1 through 9 at all sites, with the greatest difference between cycles 1 and 2 particularly in the superficial layer (*p* < 0.001, Supplementary Figure 4.1).

There were significant associations between BVTV and hysteresis at all sites, however, the nature of these associations differed between sites (Supplementary Figure 4.2). In the palmar MCIII, hysteresis decreased quadratically with higher BVTV (*p* < 0.001). In the dorsal MCIII, specimens with higher BVTV had greater hysteresis, and the magnitude of effect on hysteresis decreased with higher BVTV (*p* = 0.002). In the proximal sesamoid, BVTV was associated with greater hysteresis and the effect of BVTV was greater in the superficial layer (*p* < 0.001).

Bone mineral density was quadratically associated with hysteresis at all sites, with hysteresis decreasing with increasing BMD, and the magnitude of this effect was greatest for the dorsal MCIII (*p* = 0.028), followed by palmar MCIII (*p* < 0.001), then sesamoid (*p* < 0.001, Supplementary Figure 4.3).

In the palmar MCIII site, greater hysteresis was associated with specimens from right forelimbs compared to the left forelimbs (*p* < 0.001), horses euthanized due to a catastrophic musculoskeletal injury compared to those that died or were euthanized for other reasons (*p* < 0.001), specimens with thicker cartilage (*p* < 0.001), and specimens with a more convex mineralized tissue surface (Even A, *p* < 0.001). In the proximal sesamoid, hysteresis was greater in older horses (*p* = 0.029).

## Discussion

In this study, we examined the structural and mechanical properties of SCB and cartilage specimens from three different sites and at two depths in the fetlock joint of Thoroughbred racehorses. We found that the dorsal MCIII SCB has lower BVTV, BMD, and stiffness compared to the palmar MCIII and sesamoid bone. Superficial SCB had higher BVTV and lower BMD than deeper SCB except at the palmar MCIII site where there was little difference in BVTV between depths, and at all sites, the deep bone was stiffer. Hysteresis was greater superficially in palmar MCIII and sesamoid, but not dorsal MCIII specimens. For whole cartilage-bone samples, stiffness increased and hysteresis decreased with cyclic loading, however, no change in stiffness was observed in the individual layers.

Higher BVTV, BMD, and stiffness in the palmar MCIII and sesamoid sites agree with our hypothesis and are likely an adaptation to the greater stress experienced by these sites relative to the dorsal MCIII during galloping (24). In our multivariable model, lower BVTV in the dorsal MCIII largely explains the lower stiffness. High stress on unadapted bone induces high strain. Beyond an upper threshold, strain drives bone modeling and an increase in BVTV (30). As the palmar MCIII and sesamoid bones articulate and are under similar compressive force, it is understandable their structure would be similar (31). Our palmar MCIII BVTV results are slightly lower and more variable than those previously published, while our BMD results are higher and more variable (16). This was partially explained by a low outlier in our data set with a BVTV of only 0.70 superficially. The differences in BVTV and BMD between studies may represent densification and remodeling due to different training volumes and microdamage burdens**. **Our samples were selected to have relatively low damage burdens and were therefore likely to have lower BVTV as damage and BVTV are highly correlated (9, 13). Increasing bone mineral density with increased distance from the articular surface is a consistent feature in the palmar MCIII bone (16, 20, 32) and was also present in our dorsal MCIII and proximal sesamoid specimens.

To our knowledge, the microstructure of the proximal sesamoid or dorsal MCIII bone has not been examined using our methodology. Our total specimen proximal sesamoid bone volume fraction results are comparable to a study isolating the midbody dorsal sesamoid bone (mean bone volume fraction 0.86 ± 0.06, similar to our 0.83 ± 0.05); however the bone mineral density in our specimens appears higher (mean 928.29 ± 12.60 compared to 828.56 ± 19.60 mg HA/ccm) (13). This may be due to differences in site selection, resolution of imaging, or local or individual microdamage as a number of microfractures are associated with BVTV in the proximal sesamoid (13). In the dorsal MCIII bone, a mean bone volume fraction of 0.73 ± 0.08 has been reported, similar to the 0.71 ± 0.08 we observed (15).

While overall, the initial stiffness of palmar MCIII in our study (3007.20 ± 523.80 MPa) was comparable to others (2.362 ± 443 to 2,904.7 ± 112.8 MPa) (17, 19, 20, 28), Malekipour et al. (16) assessed equivalent layers in our study and reported greater total (5608.3 ± 2407.6) and lower superficial (2446.4 ± 1132.8 MPa) and deep (6890.4 ± 1741.1 MPa) stiffness. These differences between studies are likely due to varying microdamage burdens [in the previous study, two specimens had microfractures and both failed at 44.2 ± 10.7 MPa stress (2.33 ± 0.25% strain)] and microstructure. Strain rate influences stiffness, therefore the greater strain rate used by Malekipour et al. (16) could also contribute to their greater total cartilage-bone specimen stiffness (33). Additionally, differences in materials testing device and digital image correlation technique may partially explain differences in stiffness between studies (16). To the best of our knowledge, no data exist on dorsal MCIII or proximal sesamoid bone biomechanics.

At all sites, stiffness was greater in the deep, relative to the superficial SCB. This finding has been reported for single impact loading of palmar MCIII bone, a site prone to SCB damage in Thoroughbred racehorses, and the magnitude of this gradient was not explained by differences in BVTV or BMD between regions; rather, it was thought that microdamage below the limit of detection by microCT was largely responsible for lower stiffness and greater hysteresis in the superficial layer (16). We showed that this stiffness gradient is also present at a site where SCB damage is rare although the magnitude of the gradient in our study was lower in dorsal MCIII bone, mainly due to lower stiffness in the deep layer. This lower stiffness in the dorsal MCIII deep layer is largely explained by lower BVTV and BMD than the equivalent palmar MCIII and sesamoid layers. Superficial stiffness, however, does not differ nearly as much between sites, yet there are still large differences in structure in that layer (BVTV and BMD are lower dorsally). It is likely that bone modeling does not compensate for greater microdamage in the palmar MCIII and sesamoid superficial layer. However in the deeper bone, modeling in the absence of microdamage increases stiffness resulting in a greater stiffness gradient at these sites than at the dorsal bone.

Microdamage is a good predictor of low stiffness and high hysteresis (17). In our study, adjusted damaged bone volume fraction was not associated with stiffness in all sites and layers. Where associations did exist, they differed: in the sesamoid, we observed greater stiffness, while in the deep dorsal MCIII, we observed lower stiffness in association with more microdamage. It is possible that at the relatively low microdamage levels observed in our samples, BVTV and BMD had a greater influence on bone resilience than samples with greater damage burdens. The damage we did identify was predominantly within 1.0 mm of the mineralized tissue surface or the most superficial part of the superficial region of interest, thus playing only a minor role in the properties of this region. In addition, the technique we used for microdamage quantification is not highly specific. The lead-uranyl acetate complex can stain osteoid seams and canaliculi of osteocytes, and higher resolution imaging would be required to differentiate these from microdamage (34). Superficially, damaged bone volume was greater in the sesamoid site, compared to the palmar MCIII site. This was unexpected because unstained microdamage and staining that followed a pattern consistent with microcracking were only observed in the palmar MCIII site. If not caused by microdamage, a possible explanation for the increased uptake seen in the proximal sesamoid is different trabecular architecture in these specimens allowing greater penetration of the stain. Hysteresis may be a better indicator of fatigue damage accumulation, as in the dorsal MCIII site deep and superficial hysteresis did not differ.

In our study, we found no evidence that the superficial and deep layers contribute to the whole sample's increased stiffness with cyclic loading. This lack of an increase in stiffness with cycles in the superficial and deep layers may be due to several factors. The number of superficial and deep cycles analyzed (cycles 1, 2, 3, 5, and 9) was limited compared to total cartilage-bone specimens due to software and camera memory constraints. Superficial and deep SCB stiffness was also more variable than total cartilage-bone specimen stiffness, likely due to the nature of digital image correlation evaluating one 2-dimensional surface of a 3-dimensional structure. Leaving cartilage *in situ* was responsible for a significant percentage of whole specimen initial stiffening; articular cartilage compresses, stiffens at low loading magnitudes, and may take longer to reach a steady state than bone (35, 36). The severed proximal trabeculae could also cause end-artifacts. We observed an increase in whole specimen stiffness over cycles 1–99. This is consistent with previous work on palmar MCIII specimens without overlying cartilage (19, 20).

In contrast to stiffness, normalized hysteresis (energy dissipation) decreased with cyclic loading in the superficial and deep layers. Creep behavior in trabecular bone is both recoverable and non-recoverable even at low strain, with non-recoverable strains likely due to localized irreversible deformations during initial loading cycles (37). The lack of difference between superficial and deep hysteresis in the dorsal MCIII supports the theory that high shock absorption in the proximal sesamoid and palmar MCIII superficial layer is due to microdamage. Furthermore, hysteresis was associated with fracture as a cause of death in the palmar MCIII. Bone mineral density, architecture, or mineral crystal size could also contribute to shock absorption behavior (38, 39).

This study shows that the biomechanical behavior of SCB throughout the fetlock joint is complex. More associations were found between structure and mechanics in our cyclic loading study than in a single impact design (16). However, biomechanical behavior was still only partially explained by SCB density, and the nature of associations–when present–can vary between sites within the same joint. While whole sample mechanical properties and properties of bone depths at each site were consistent with bone density measurement (dorsal site less dense and less stiff compared to the palmar site for whole bone samples and when comparing each depth) this is not the case when comparing superficial and deep sites. For example, at the dorsal site, the superficial bone had a 12% higher apparent density (BVTV × BMD) than the deeper bone yet had 28% lower stiffness and at the palmar site, the apparent density was similar at each depth, yet the superficial bone was 41% less stiff than the deep bone. The gradient of stiffness from superficial to deeper bone was consistent throughout all three sites despite differences in bone density and microdamage levels suggesting this is an important property of SCB, which likely maintains joint congruity under loading and minimizes abrupt changes in stiffness from the articular surface through to the stiff deeper layers.

As bone is an organic-inorganic composite, there are many micro- and ultra-structural facets that likely contribute to the specialized properties of the superficial SCB layer (38). The lower BMD superficially is at least partly due to a higher remodeling rate as lower BMD is seen in newly remodeled bone (9, 40, 41). Higher bone turnover in the superficial layer may have additional effects, such as changes in microarchitecture, collagen network orientation, and crosslinking. Although we did not observe good evidence of decreased stiffness in association with microdamage, others have shown that small amounts of microdamage have a considerable effect on stiffness (42–44), therefore it is possible that damage that we were unable to accurately quantify with our methodology contributes to the lower superficial stiffness.

The superficial and deep zones did not appear to contribute to the increase in stiffness of whole cartilage and bone samples with cyclic loading that we observed. This phenomenon is likely due to residual strain accumulation and creep deformation at sites within the specimens that were not monitored with digital image correlation. Changes in hysteresis of whole samples were reflected in the superficial and deep SCB layers. The superficial layer seems to play an important role in shock absorption which persists despite some reduction with cyclic loading.

Our specimen collection technique allowed examination of only small sections of bone from each site, however examining a larger field of view would make later strain evaluation problematic and increase microCT scan time markedly if a similar spatial resolution were to be maintained. The mineralized cartilage layer was not specifically assessed because it was obscured from view by the non-mineralized cartilage during loading, making the digital image correlation of this layer inaccurate. Our local strain data was more variable than total cartilage-bone specimen data, highlighting an inherent limitation of 2-dimensional evaluation of a 3-dimensional non-uniform biological structure. The lead-uranyl acetate staining technique could only be used at one time point so we were unable to determine how much label uptake was due to the *in vivo* accumulation of microdamage or staining artifacts, and how much was due to *ex vivo* mechanical testing (45). In addition, back scattered electron microscopy on some specimens may have differentiated between microdamage, osteoid seam, and canaliculi uptake, and whether this varied across sites. There is currently no method of non-destructively quantifying diffuse microdamage prior to *ex vivo* loading. The use of unconfined specimens during testing does not reproduce *in vitro* loading and has its own inherent limitations; however, this technique is the standard for bone testing to facilitate comparison between studies (46).

## Conclusion

This study highlights the complex behavior of SCB underloading in the fetlock joint of equine athletes. Superficial bone was consistently less stiff than deeper bone despite differences in bone density and damage levels between sites. This more compliant superficial layer may be important for joint physiology. The superficial layer is also important for energy dissipation at the sites subjected to the highest loads in the galloping horse but whether this is a specific adaptation or a result of microdamage accumulation is not clear. Future work should investigate how the superficial SCB achieves its specialized role in joint biomechanics.

## Data Availability Statement

The raw data supporting the conclusions of this article will be made available by the authors, without undue reservation.

## Ethics Statement

The animal study was reviewed and approved by the University of Melbourne Animal Ethics Committee. Written informed consent was obtained from the owners for the participation of their animals in this study.

## Author Contributions

DP, FM, PL, and RCW: study conception and design. DP and BA: investigation and acquisition of data. DP, PH, and RCW: data analysis and interpretation. DP: drafting of the manuscript. DP and RCW: acquisition of funding. RCW and PH: supervision of the project. All authors contributed to and approved the final manuscript.

## Funding

This work was supported by Racing Australia, Racing Victoria Limited, the Victorian Racing Industry Fund of the Victorian State Government, and the University of Melbourne. Funding sources had no role in study design, interpretation, or publication.

## Conflict of Interest

The authors declare that the research was conducted in the absence of any commercial or financial relationships that could be construed as a potential conflict of interest.

## Publisher's Note

All claims expressed in this article are solely those of the authors and do not necessarily represent those of their affiliated organizations, or those of the publisher, the editors and the reviewers. Any product that may be evaluated in this article, or claim that may be made by its manufacturer, is not guaranteed or endorsed by the publisher.

## Abbreviations

MCIII: third metacarpal condyle
SCB: subchondral bone
BVTV: bone volume fraction
BMD: bone mineral density.

## References

[1] Wylie CE McManus P McDonald C Jorgensen S McGreevy P. Thoroughbred fatality and associated jockey falls and injuries in races in New South Wales and the Australian Capital Territory, Australia: 2009-2014. Vet J. (2017) 227:1–7. 10.1016/j.tvjl.2017.06.008 29031324

[2] Rosanowski SM Chang YM Stirk AJ Verheyen KLP. Epidemiology of race-day distal limb fracture in flat racing thoroughbreds in Great Britain (2000-2013). Equine Vet J. (2019) 51:83–9. 10.1111/evj.12968 29806972

[3] Sun TC Riggs CM Cogger N Wright J Al-Alawneh JI. Noncatastrophic and catastrophic fractures in racing thoroughbreds at the Hong Kong Jockey Club. Equine Vet J. (2019) 51:77–82. 10.1111/evj.12953 29672909

[4] Spargo KE Rubio-Martinez LM Wheeler DP Fletcher L Carstens A. Catastrophic musculoskeletal injuries in thoroughbred racehorses on racetracks in Gauteng, South Africa. J S Afr Vet Assoc. (2019) 90:5. 10.4102/jsava.v90i0.1640 30843400 PMC7081728

[5] Parkin IDH Clegg PD French NP Proudman CJ Riggs CM Singer ER. Risk of fatal distal limb fractures among thoroughbreds involved in the five types of racing in the United Kingdom. Vet Rec. (2004) 154:493–7. 10.1136/vr.154.16.493 15130054

[6] Johnson BJ Stover SM Daft BM Kinde H Read DH Barr BC. Causes of death in racehorses over a 2-year period. Equine Vet J. (1994) 26:327–30. 10.1111/j.2042-3306.1994.tb04395.x 8575402

[7] Norrdin RW Stover SM. Subchondral bone failure in overload arthrosis: a scanning electron microscopic study in horses. J Musculoskelet Neuronal Interact. (2006) 6:251–7. 17142946

[8] Hassan EB Mirams M Mackie EJ Whitton RC. Prevalence of subchondral bone pathological changes in the distal metacarpi/metatarsi of racing thoroughbred horses. Aust Vet J. (2017) 95:362–9. 10.1111/avj.12628 28948629

[9] Whitton RC Ayodele BA Hitchens PL Mackie EJ. Subchondral bone microdamage accumulation in distal metacarpus of thoroughbred racehorses. Equine Vet J. (2018) 50:766–73. 10.1111/evj.12948 29660153

[10] Harrison SM Whitton RC Kawcak CE Stover SM Pandy MG. Relationship between muscle forces, joint loading and utilization of elastic strain energy in equine locomotion. J Exp Biol. (2010) 213:3998–4009. 10.1242/jeb.044545 21075941

[11] Pinchbeck GL Clegg PD Boyde A Riggs CM. Pathological and clinical features associated with palmar/plantar osteochondral disease of the metacarpo/metatarsophalangeal joint in thoroughbred racehorses. Equine Vet J. (2013) 45:587–92. 10.1111/evj.12036 23418959

[12] Trope GD Anderson GA Whitton RC. Patterns of scintigraphic uptake in the fetlock joint of Thoroughbred racehorses and the effect of increased radiopharmaceutical uptake in the distal metacarpal/tarsal condyle on performance. Equine Vet J. (2011) 43:509–15. 10.1111/j.2042-3306.2010.00316.x 21545647

[13] Ayodele BA Hitchens PL Wong ASM Mackie EJ Whitton RC. Microstructural properties of the proximal sesamoid bones of Thoroughbred racehorses in training. Equine Vet J. (2021) 53:1169-77. 10.1111/evj.13394 33244781

[14] Norrdin RW Kawcak CE Capwell BA McIlwraith CW. Subchondral bone failure in an equine model of overload arthrosis. Bone. (1998) 22:133–9. 10.1016/S8756-3282(97)00253-6 9477236

[15] Riggs CM Whitehouse GH Boyde A. Structural variation of the distal condyles of the third metacarpal and third metatarsal bones in the horse. Equine Vet J. (1999) 31:130–9. 10.1111/j.2042-3306.1999.tb03806.x 10213425

[16] Malekipour F Whitton CR Lee PVS. Stiffness and energy dissipation across the superficial and deeper third metacarpal subchondral bone in Thoroughbred racehorses under high-rate compression. J Mech Behav Biomed Mater. (2018) 85:51–6. 10.1016/j.jmbbm.2018.05.031 29852352

[17] Malekipour F Hitchens PL Whitton RC Lee PVS. Effects of in vivo fatigue-induced subchondral bone microdamage on the mechanical response of cartilage-bone under a single impact compression. J Biomech. (2020) 100:11. 10.1016/j.jbiomech.2019.109594 31924348

[18] Shaktivesh S Malekipour F Whitton RC Hitchens PL Lee PV. Fatigue behavior of subchondral bone under simulated physiological loads of equine athletic training. J Mech Behav Biomed Mater. (2020) 110:10. 10.1016/j.jmbbm.2020.103920 32957215

[19] Martig S Lee PVS Anderson GA Whitton RC. Compressive fatigue life of subchondral bone of the metacarpal condyle in thoroughbred racehorses. Bone. (2013) 57:392–8. 10.1016/j.bone.2013.09.006 24063945

[20] Martig S Hitchens PL Lee PVS Whitton RC. The relationship between microstructure, stiffness and compressive fatigue life of equine subchondral bone. J Mech Behav Biomed Mater. (2019) 101:103439. 10.1016/j.jmbbm.2019.103439 31557658

[21] Linde F Hvid I Jensen NC. Material properties of cancellous bone in repetitive axial loading. Eng Med. (1985) 14:173–7. 10.1243/EMED_JOUR_1985_014_042_02 4092810

[22] Hassan EB Mirams M Ghasem-Zadeh A Mackie EJ Whitton RC. Role of subchondral bone remodelling in collapse of the articular surface of Thoroughbred racehorses with palmar osteochondral disease. Equine Vet J. (2016) 48:228–33. 10.1111/evj.12415 25582246

[23] Schindelin J Arganda-Carreras I Frise E Kaynig V Longair M Pietzsch T. Fiji: an open-source platform for biological-image analysis. Nat Methods. (2012) 9:676–82. 10.1038/nmeth.2019 22743772 PMC3855844

[24] Harrison SM Whitton RC Kawcak CE Stover SM Pandy MG. Evaluation of a subject-specific finite-element model of the equine metacarpophalangeal joint under physiological load. J Biomech. (2014) 47:65–73. 10.1016/j.jbiomech.2013.10.001 24210848

[25] Martig S. Compressive fatigue life and micromorphology of equine metacarpal subchondral bone (PhD thesis). In: Faculty of Veterinary and Agricultural Sciences and Melbourne School of Engineering, Department of Biomedical Engineering. The University of Melbourne, Melbourne (2019).

[26] Lambers FM Bouman AR Rimnac CM Hernandez CJ. Microdamage caused by fatigue loading in human cancellous bone: relationship to reductions in bone biomechanical performance. PLoS ONE. (2013) 8:e0083662. 10.1371/journal.pone.0083662 24386247 PMC3875472

[27] Faul F Erdfelder E Lang AG Buchner A. G^*^Power 3: a flexible statistical power analysis program for the social, behavioral, and biomedical sciences. Behav Res Methods. (2007) 39:175–91. 10.3758/BF03193146 17695343

[28] Rubio-Martinez LM Cruz AM Gordon K Hurtig MB. Mechanical properties of subchondral bone in the distal aspect of third metacarpal bones from Thoroughbred racehorses. Am J Vet Res. (2008) 69:1423–33. 10.2460/ajvr.69.11.1423 18980424

[29] Cresswell EN McDonough SP Palmer SE Hernandez CJ Reesink HL. Can quantitative computed tomography detect bone morphological changes associated with catastrophic proximal sesamoid bone fracture in Thoroughbred racehorses? Equine Vet J. (2019) 51:123–30. 10.1111/evj.12965 29758110

[30] Frost HM. A 2003 update of bone physiology and Wolff's Law for clinicians. Angle Orthod. (2004) 74:3–15. 10.1043/0003-3219(2004)074<0003:AUOBPA>2.0.CO;2 15038485

[31] Thomason JJ. The relationship of structure to mechanical function in the 3rd metacarpal bone of the horse, equus-caballus. Can J Zool. (1985) 63:1420–8. 10.1139/z85-212

[32] Martig S Hitchens PL Stevenson MA Whitton RC. Subchondral bone morphology in the metacarpus of racehorses in training changes with distance from the articular surface but not with age. J Anat. (2018) 232:919–30. 10.1111/joa.12794 29446086 PMC5979622

[33] Linde F Norgaard P Hvid I Odgaard A Soballe K. Mechanical-properties of trabecular bone - dependency on strain rate. J Biomech. (1991) 24:803–9. 10.1016/0021-9290(91)90305-7 1752864

[34] Schaffler MB Pitchford WC Choi K Riddle JM. Examination of compact-bone microdamage using backscattered electron-microscopy. Bone. (1994) 15:483–8. 10.1016/8756-3282(94)90271-2 7526878

[35] Kaplan JT Neu CP Drissi H Emery NC Pierce DM. Cyclic loading of human articular cartilage: The transition from compaction to fatigue. J Mech Behav Biomed Mater. (2017) 65:734–42. 10.1016/j.jmbbm.2016.09.040 27756049

[36] Rohl L Linde F Odgaard A Hvid I. Simultaneous measurement of stiffness and energy absorptive properties of articular cartilage and subchondral trabecular bone. Proc Inst Mech Eng H. (1997) 211:257–64. 10.1243/0954411971534368 9256002

[37] Manda K Wallace RJ Xie SQ Levrero-Florencio F Pankaj P. Nonlinear viscoelastic characterization of bovine trabecular bone. Biomech Model Mechanobiol. (2017) 16:173–89. 10.1007/s10237-016-0809-y 27440127 PMC5285425

[38] Luo Y Wu X. Bone quality is dependent on the quantity and quality of organic–inorganic phases. J Med Biol Eng. (2020) 40:273–81. 10.1007/s40846-020-00506-x

[39] Bala Y Seeman E. Bone's material constituents and their contribution to bone strength in health, disease, and treatment. Calcif Tissue Int. (2015) 97:308–26. 10.1007/s00223-015-9971-y 25712256

[40] Burr DB. The importance of subchondral bone in the progression of osteoarthritis. J Rheumatol. (2004) 31:77–80. 15132360

[41] Boyde A Firth EC. Musculoskeletal responses of 2-year-old Thoroughbred horses to early training. 8 Quantitative back-scattered electron scanning electron microscopy and confocal fluorescence microscopy of the epiphysis of the third metacarpal bone. N Z Vet J. (2005) 53:123–32. 10.1080/00480169.2005.36489 15846396

[42] Hernandez CJ Lambers FM Widjaja J Chapa C Rimnac CM. Quantitative relationships between microdamage and cancellous bone strength and stiffness. Bone. (2014) 66:205–13. 10.1016/j.bone.2014.05.023 24928495 PMC4125443

[43] Burr DB Turner CH Naick P Forwood MR Ambrosius W Hasan MS. Does microdamage accumulation affect the mechanical properties of bone? J Biomech. (1998) 31:337–45. 10.1016/S0021-9290(98)00016-5 9672087

[44] Seref-Ferlengez Z Kennedy OD Schaffler MB. Bone microdamage, remodeling and bone fragility: how much damage is too much damage? Bonekey Rep. (2015) 4. 10.1038/bonekey.2015.11 25848533 PMC4371415

[45] Tang SY Vashishth D. A non-invasive in vitro technique for the three-dimensional quantification of microdamage in trabecular bone. Bone. (2007) 40:1259–64. 10.1016/j.bone.2006.10.031 17329178 PMC3312747

[46] Zhao S Arnold M Ma S Abel RL Cobb JP Hansen U. Standardizing compression testing for measuring the stiffness of human bone. Bone Joint Res. (2018) 7:524–38. 10.1302/2046-3758.78.BJR-2018-0025.R1 30258572 PMC6138811

