# Supplementary material for: Biomechanical and Microstructural Properties of Subchondral Bone From Three Metacarpophalangeal Joint Sites in Thoroughbred Racehorses
Source: Front Vet Sci. 2022 Jun 28;9:923356. doi: 10.3389/fvets.2022.923356 (PMC9277662; doi:10.3389/fvets.2022.923356)
Supplement: Supplementary file 1 [file Data_Sheet_1.zip › Supplementary Item 1.DOCX]

Supplementary Item 1. Case details and palmar osteochondral disease grading system.

**Table 1.1.** Case description data for the n = 10 Thoroughbred racehorses used in this study. Side = forelimb from which specimens were obtained; F = female, ME = male entire, MN = gelding; POD grade = palmar osteochondral disease grade.

| Case number | Age (years) | Side | Sex | POD grade | Cause of death |
| --- | --- | --- | --- | --- | --- |
| 1 | 3 | Left | F | 0 | Sudden death |
| 2 | 3 | Right | ME | 0 | Hind pastern fracture |
| 3 | 3 | Right | MN | 0 | Sudden death |
| 4 | 4 | Left | ME | 1 | Sudden death |
| 5 | 3 | Left | F | 0 | Pelvic fracture |
| 6 | 4 | Right | F | 1 | Humeral fracture |
| 7 | 3 | Right | MN | 0 | Pulmonary edema |
| 8 | 3 | Right | MN | 0 | Sudden death |
| 9 | 3 | Right | ME | 1 | Radial fracture |
| 10 | 3 | Right | F | 1 | Pelvic fracture |

**Table 1.2.** Scoring system for gross pathology of the disto-palmar third metacarpal condyles. Adapted from (1).

| **Palmar osteochondral disease (POD) grade** | **Description** |
| --- | --- |
| 0 | Normal |
| 1 | Discolouration (bruising) of subchondral bone with no or minimal disruption of the overlying articular cartilage |
| 2 | Discolouration, with mild to moderate disruption of articular cartilage |
| 3 | Discolouration and disruption or collapse of articular surface |

1. G. L. Pinchbeck, P. D. Clegg, A. Boyde and C. M. Riggs: Pathological and clinical features associated with palmar/plantar osteochondral disease of the metacarpo/metatarsophalangeal joint in Thoroughbred racehorses. *Equine Veterinary Journal*, 45(5), 587-592 (2013) doi:10.1111/evj.12036
