# Supplementary material for: Biomechanical and Microstructural Properties of Subchondral Bone From Three Metacarpophalangeal Joint Sites in Thoroughbred Racehorses
Source: Front Vet Sci. 2022 Jun 28;9:923356. doi: 10.3389/fvets.2022.923356 (PMC9277662; doi:10.3389/fvets.2022.923356)
Supplement: Supplementary file 1 [file Data_Sheet_1.zip › Supplementary Item 2.DOCX]

Supplementary Item 2. Univariable associations between site, depth and microstructure.

**Table 2.1.** Bone volume fraction of subchondral bone specimens from the metacarpophalangeal joint of n = 10 Thoroughbred racehorses with means, standard deviations (s.d.), and univariable mixed effects linear model estimated regression coefficients (Coef.), their 95% confidence intervals, and alpha level (P-value) of bone volume fraction and site (dorsal MCIII, palmar MCIII, or proximal sesamoid) associations at various depths of the bone (superficial 2 mm, deeper 2 mm and total ~10 mm thick specimens).

| **Variable** | **Mean (s.d.)** | **Coef.** | **95% Confidence Interval** | | **P-value** |
| --- | --- | --- | --- | --- | --- |
|  |  |  | **Lower**  **Bound** | **Upper**  **Bound** |  |
| Total |  |  |  |  |  |
| Sesamoid | 0.83 (0.05) | 0.13 | 0.06 | 0.20 | 0.001 |
| Palmar | 0.87 (0.10) | 0.16 | 0.09 | 0.23 | <0.001 |
| Dorsal | 0.71 (0.08) | Reference |  |  |  |
| Superficial |  |  |  |  |  |
| Sesamoid | 0.94 (0.04) | 0.13 | 0.06 | 0.20 | 0.001 |
| Palmar | 0.94 (0.09) | 0.14 | 0.06 | 0.21 | 0.001 |
| Dorsal | 0.80 (0.10) | Reference |  |  |  |
| Deep |  |  |  |  |  |
| Sesamoid | 0.85 (0.05) | 0.18 | 0.10 | 0.25 | <0.001 |
| Palmar | 0.91 (0.10) | 0.23 | 0.15 | 0.30 | <0.001 |
| Dorsal | 0.68 (0.08) | Reference |  |  |  |

**Table 2.2.** Bone volume fraction of subchondral bone specimens from the metacarpophalangeal joint of n = 10 Thoroughbred racehorses with means, standard deviations (s.d.), and univariable mixed effects linear model estimated regression coefficients (Coef.), their 95% confidence intervals, and alpha level (P-value) of bone volume fraction and layer (superficial 2 mm or deeper 2 mm) associations at various sites within the joint (dorsal MCIII, palmar MCIII, or proximal sesamoid).

| **Variable** | **Mean (s.d.)** | **Coef.** | **95% Confidence Interval** | | **P-value** |
| --- | --- | --- | --- | --- | --- |
|  |  |  | **Lower**  **Bound** | **Upper**  **Bound** |  |
| Sesamoid |  |  |  |  |  |
| Deep | 0.85 (0.05) | -0.08 | -0.13 | -0.04 | 0.001 |
| Superficial | 0.94 (0.04) | Reference |  |  |  |
| Palmar |  |  |  |  |  |
| Deep | 0.91 (0.10) | -0.03 | -0.12 | 0.05 | 0.419 |
| Superficial | 0.94 (0.09) | Reference |  |  |  |
| Dorsal |  |  |  |  |  |
| Deep | 0.68 (0.08) | -0.13 | -0.21 | -0.04 | 0.006 |
| Superficial | 0.80 (0.10) | Reference |  |  |  |

**Table 2.3.** Bone mineral density [mg HA/ccm] of subchondral bone specimens from the metacarpophalangeal joint of n = 10 Thoroughbred racehorses with means, standard deviations (s.d.), and univariable mixed effects linear model estimated regression coefficients (Coef.), their 95% confidence intervals, and alpha level (P-value) of bone mineral density and site (dorsal MCIII, palmar MCIII, or proximal sesamoid) associations at various depths of the bone (superficial 2 mm, deeper 2 mm and total ~10 mm thick specimens).

| **Variable** | **Mean (s.d.)** | **Coef.** | **95% Confidence Interval** | | **P-value** |
| --- | --- | --- | --- | --- | --- |
|  |  |  | **Lower**  **Bound** | **Upper**  **Bound** |  |
| Total |  |  |  |  |  |
| Sesamoid | 928.29 (12.60) | 37.19 | 16.13 | 58.25 | 0.001 |
| Palmar | 913.29 (22.66) | 22.19 | 1.14 | 43.25 | 0.040 |
| Dorsal | 891.10 (30.13) | Reference |  |  |  |
| Superficial |  |  |  |  |  |
| Sesamoid | 906.62 (16.24) | 50.41 | 28.58 | 72.24 | <0.001 |
| Palmar | 882.34 (28.44) | 26.13 | 4.30 | 47.96 | 0.021 |
| Dorsal | 856.21 (25.02) | Reference |  |  |  |
| Deep |  |  |  |  |  |
| Sesamoid | 945.54 (14.00) | 46.83 | 25.75 | 67.90 | <0.001 |
| Palmar | 921.86 (25.40) | 23.15 | 2.07 | 44.22 | 0.033 |
| Dorsal | 898.71 (27.24) | Reference |  |  |  |

**Table 2.4.** Bone mineral density [mg HA/ccm] of subchondral bone specimens from the metacarpophalangeal joint of n = 10 Thoroughbred racehorses with means, standard deviations (s.d.), and univariable mixed effects linear model estimated regression coefficients (Coef.), their 95% confidence intervals, and alpha level (P-value) of bone mineral density and layer (superficial 2 mm or deeper 2 mm) associations at various sites within the joint (dorsal MCIII, palmar MCIII, or proximal sesamoid).

| **Variable** | **Mean (s.d.)** | **Coef.** | **95% Confidence Interval** | | **P-value** |
| --- | --- | --- | --- | --- | --- |
|  |  |  | **Lower**  **Bound** | **Upper**  **Bound** |  |
| Sesamoid |  |  |  |  |  |
| Deep | 945.54 (14.00) | 38.92 | 24.67 | 53.16 | <0.001 |
| Superficial | 906.62 (16.24) | Reference |  |  |  |
| Palmar |  |  |  |  |  |
| Deep | 921.86 (25.40) | 39.52 | 14.19 | 64.85 | 0.004 |
| Superficial | 882.34 (28.44) | Reference |  |  |  |
| Dorsal |  |  |  |  |  |
| Deep | 898.71 (27.24) | 42.50 | 17.93 | 67.07 | 0.002 |
| Superficial | 856.21 (25.02) | Reference |  |  |  |

**Table 2.5.** Damaged bone volume fraction [damaged bone volume mm^3^ / bone volume mm^3^] of subchondral bone specimens from the metacarpophalangeal joint of Thoroughbred racehorses (n = 10 except for the deep dorsal site where n = 9) with means, standard deviations (s.d.), and univariable mixed effects linear model estimated regression coefficients (Coef.), their 95% confidence intervals, and alpha level (P-value) of damaged bone volume fraction and site (dorsal MCIII, palmar MCIII, or proximal sesamoid) associations at various depths of the bone (superficial 2 mm and deeper 2 mm of each specimen).

| **Variable** | **Number** | **Mean (s.d.)** | **Coef.** | **95% Confidence Interval** | | **P-value** |
| --- | --- | --- | --- | --- | --- | --- |
|  |  |  |  | **Lower**  **Bound** | **Upper**  **Bound** |  |
| Superficial |  |  |  |  |  |  |
| Dorsal | 10 | 0.03 (0.01) | -0.01 | -0.02 | -0.00008 | 0.049 |
| Palmar | 10 | 0.02 (0.01) | -0.02 | -0.03 | -0.01 | 0.001 |
| Sesamoid | 10 | 0.04 (0.01) | Reference |  |  |  |
| Deep |  |  |  |  |  |  |
| Dorsal | 9 | 0.01 (0.006) | -0.02 | -0.02 | -0.007 | 0.003 |
| Palmar | 10 | 0.02 (0.01) | -0.008 | -0.02 | 0.002 | 0.125 |
| Sesamoid | 10 | 0.03 (0.01) | Reference |  |  |  |

**Table 2.6.** Damaged bone volume fraction [damaged bone volume mm^3^ / bone volume mm^3^] of subchondral bone specimens from the metacarpophalangeal joint of Thoroughbred racehorses (n = 10 except for the deep dorsal site where n = 9) with means, standard deviations (s.d.), and univariable mixed effects linear model estimated regression coefficients (Coef.), their 95% confidence intervals, and alpha level (P-value) of damaged bone volume fraction and layer (superficial 2 mm and deeper 2 mm) associations at various sites within the joint (dorsal MCIII, palmar MCIII, or proximal sesamoid).

| **Variable** | **Number** | **Mean (s.d.)** | **Coef.** | **95% Confidence Interval** | | **P-value** |
| --- | --- | --- | --- | --- | --- | --- |
|  |  |  |  | **Lower**  **Bound** | **Upper**  **Bound** |  |
| Sesamoid |  |  |  |  |  |  |
| Deep | 10 | 0.03 (0.01) | -0.006 | -0.02 | 0.006 | 0.277 |
| Superficial | 10 | 0.04 (0.01) | Reference |  |  |  |
| Palmar |  |  |  |  |  |  |
| Deep | 10 | 0.02 (0.01) | 0.008 | -0.005 | 0.02 | 0.212 |
| Superficial | 10 | 0.02 (0.01) | Reference |  |  |  |
| Dorsal |  |  |  |  |  |  |
| Deep | 9 | 0.01 (0.006) | -0.01 | -0.02 | -0.002 | 0.026 |
| Superficial | 10 | 0.03 (0.01) | Reference |  |  |  |

**Table 2.7.** Adjusted damaged bone volume fraction [damaged bone volume fraction / bone surface area (mm^-2^)] of subchondral bone specimens from the metacarpophalangeal joint of Thoroughbred racehorses (n = 10 except for the deep dorsal site where n = 9) with means, standard deviations (s.d.), and univariable mixed effects linear model estimated regression coefficients (Coef.), their 95% confidence intervals, and alpha level (P-value) of adjusted bone volume fraction and site (dorsal MCIII, palmar MCIII, or proximal sesamoid) associations at various depths of the bone (superficial 2 mm and deeper 2 mm of each specimen).

| **Variable** | **Number** | **Mean (s.d.)** | **Coef.** | **95% Confidence Interval** | | **P-value** |
| --- | --- | --- | --- | --- | --- | --- |
|  |  |  |  | **Lower**  **Bound** | **Upper**  **Bound** |  |
| Superficial |  |  |  |  |  |  |
| Dorsal | 10 | 0.0002 (0.0002) | -0.0004 | -0.0007 | -0.0002 | <0.001 |
| Palmar | 10 | 0.0003 (0.0002) | -0.0004 | -0.0006 | -0.0001 | 0.002 |
| Sesamoid | 10 | 0.0007 (0.0004) | Reference |  |  |  |
| Deep |  |  |  |  |  |  |
| Dorsal | 9 | 0.0001 (0.00003) | -0.0003 | -0.0006 | -0.000006 | 0.046 |
| Palmar | 10 | 0.0005 (0.0005) | 0.0001 | -0.0002 | 0.0004 | 0.494 |
| Sesamoid | 10 | 0.0004 (0.0002) | Reference |  |  |  |

**Table 2.8.** Adjusted damaged bone volume fraction [damaged bone volume fraction / bone surface area (mm^-2^)] of subchondral bone specimens from the metacarpophalangeal joint of Thoroughbred racehorses (n = 10 except for the deep dorsal site where n = 9 ) with means, standard deviations (s.d.), and univariable mixed effects linear model estimated regression coefficients (Coef.), their 95% confidence intervals, and alpha level (P-value) of adjusted bone volume fraction and layer (superficial 2 mm and deeper 2 mm) associations at various sites within the joint (dorsal MCIII, palmar MCIII, or proximal sesamoid).

| **Variable** | **Number** | **Mean (s.d.)** | **Coef.** | **95% Confidence Interval** | | **P-value** |
| --- | --- | --- | --- | --- | --- | --- |
|  |  |  |  | **Lower**  **Bound** | **Upper**  **Bound** |  |
| Sesamoid |  |  |  |  |  |  |
| Deep | 10 | 0.0004 (0.0002) | -0.0003 | -0.0006 | -0.000002 | 0.048 |
| Superficial | 10 | 0.0007 (0.0004) | Reference |  |  |  |
| Palmar |  |  |  |  |  |  |
| Deep | 10 | 0.0005 (0.0005) | 0.0002 | -0.0001 | 0.0005 | 0.249 |
| Superficial | 10 | 0.0003 (0.0002) | Reference |  |  |  |
| Dorsal |  |  |  |  |  |  |
| Deep | 9 | 0.0001 (0.00003) | -0.0001 | -0.0002 | -0.00002 | 0.027 |
| Superficial | 10 | 0.0002 (0.0002) | Reference |  |  |  |
