# Supplementary material for: Biomechanical and Microstructural Properties of Subchondral Bone From Three Metacarpophalangeal Joint Sites in Thoroughbred Racehorses
Source: Front Vet Sci. 2022 Jun 28;9:923356. doi: 10.3389/fvets.2022.923356 (PMC9277662; doi:10.3389/fvets.2022.923356)
Supplement: Supplementary file 1 [file Data_Sheet_1.zip › Supplementary Item 3.docx]

Supplementary Item 3. Associations between stiffness and study factors.

**Table 3.1.** Stiffness (MPa) of total cartilage-bone specimen with means, standard deviations (s.d.), and univariable mixed effects linear model estimated regression coefficients (Coef.), their 95% confidence intervals, and alpha level (P-value) of factors associated with total specimen stiffness (n = 420) of three sites (dorsal MCIII, palmar MCIII, or proximal sesamoid) within the metacarpophalangeal joint of n = 10 Thoroughbred racehorses. Adjusted for multiple measurements of site and cycle within horse to account for horse-level random effects.

| **Variable** | **Number** | **Mean (s.d.)** | **Coef.** | **95% Confidence Interval** | | **P-value** |
| --- | --- | --- | --- | --- | --- | --- |
|  |  |  |  | **Lower**  **Bound** | **Upper**  **Bound** |  |
| Cycle |  |  |  |  |  |  |
| 1 | 30 | 2632.98 (574.05) | Reference |  |  |  |
| 2 | 30 | 2930.78 (602.97) | 297.79 | 44.31 | 551.28 | 0.021 |
| 3 | 30 | 2963.65 (604.37) | 330.67 | 77.19 | 584.15 | 0.011 |
| 5 | 30 | 3001.12 (600.49) | 368.14 | 114.66 | 621.62 | 0.004 |
| 9 | 30 | 3045.20 (600.92) | 412.21 | 158.73 | 665.70 | 0.001 |
| 19 | 30 | 3105.52 (601.57) | 472.53 | 219.05 | 726.02 | <0.001 |
| 29 | 30 | 3149.69 (603.00) | 516.71 | 263.23 | 770.19 | <0.001 |
| 39 | 30 | 3183.70 (604.29) | 550.72 | 297.24 | 804.21 | <0.001 |
| 49 | 30 | 3214.79 (608.41) | 581.80 | 328.32 | 835.29 | <0.001 |
| 59 | 30 | 3243.74 (608.41) | 610.76 | 357.28 | 864.24 | <0.001 |
| 69 | 30 | 3267.90 (611.71) | 634.92 | 381.44 | 888.40 | <0.001 |
| 79 | 30 | 3292.17 (610.68) | 659.18 | 405.70 | 912.67 | <0.001 |
| 89 | 30 | 3313.86 (616.11) | 680.88 | 427.40 | 934.36 | <0.001 |
| 99 | 30 | 3333.74 (619.12) | 700.76 | 447.28 | 954.24 | <0.001 |
| Site |  |  |  |  |  |  |
| Sesamoid | 140 | 3420.02 (529.82) | Reference |  |  |  |
| Dorsal | 140 | 2532.15 (379.05) | -887.87 | -965.26 | -810.48 | <0.001 |
| Palmar | 140 | 3407.58 (475.01) | -12.44 | -89.83 | 64.95 | 0.753 |
| Sex |  |  |  |  |  |  |
| Female | 168 | 3288.04 (546.91) | Reference |  |  |  |
| Gelding | 126 | 3219.23 (672.98) | -68.81 | -444.31 | 306.69 | 0.719 |
| Entire | 126 | 2796.43 (545.31) | -491.61 | -867.11 | -116.11 | 0.010 |
| Limb |  |  |  |  |  |  |
| Right | 294 | 3173.21 (609.36) | Reference |  |  |  |
| Left | 126 | 2995.57 (640.83) | -177.65 | -609.50 | 254.21 | 0.420 |
| Fracture |  |  |  |  |  |  |
| No | 210 | 3011.54 (651.08) | Reference |  |  |  |
| Yes | 210 | 3228.30 (576.17) | 216.76 | -168.97 | 602.50 | 0.271 |
| POD |  |  |  |  |  |  |
| 0 | 252 | 3179.75 (622.55) | Reference |  |  |  |
| 1 | 168 | 3030.16 (616.04) | -149.59 | -556.04 | 256.86 | 0.471 |
| Microcrack |  |  |  |  |  |  |
| 0 | 406 | 3107.24 (630.08) | Reference |  |  |  |
| 1 | 14 | 3487.46 (115.31) | 682.97 | 350.84 | 1015.10 | <0.001 |
| Resorption |  |  |  |  |  |  |
| 1 | 294 | 3177.45 (622.35) | Reference |  |  |  |
| 0 | 126 | 2985.69 (607.85) | 125.89 | -11.33 | 263.10 | 0.072 |
|  |  |  |  |  |  |  |
| BVTV |  |  | 4890.44 | 4493.27 | 5287.62 | <0.001 |
| BMD |  |  | 18.16 | 16.210 | 20.11 | <0.001 |
| Age (months) |  |  | -32.91 | -71.06 | 5.24 | 0.091 |
| Cartilage (mm) |  |  | -2502.55 | -3271.32 | -1733.79 | <0.001 |
| Angle A |  |  | 50.17 | 15.31 | 85.03 | 0.005 |
| Angle B |  |  | 10.82 | -13.14 | 34.78 | 0.376 |
| Even A |  |  | -244.42 | -294.83 | -194.02 | <0.001 |
| Even B |  |  | -720.79 | -827.71 | -613.87 | <0.001 |

**Table 3.2.** Stiffness (MPa) of palmar MCIII subchondral bone specimens with means, standard deviations (s.d.), and univariable mixed effects linear model estimated regression coefficients (Coef.), their 95% confidence intervals, and alpha level (P-value) of factors associated with stiffness (n = 100) of the subchondral bone at two depths (superficial 2 mm or deeper 2 mm) within each specimen from the metacarpophalangeal joint of n = 10 Thoroughbred racehorses. Adjusted for depth and cycle as fixed-effect categorical variables, and for horse-level random effects.

| **Variable** | **Number** | **Mean (s.d.)** | **Coef.** | **95% Confidence Interval** | | **P-value** |
| --- | --- | --- | --- | --- | --- | --- |
|  |  |  |  | **Lower**  **Bound** | **Upper**  **Bound** |  |
| Cycle |  |  |  |  |  |  |
| 1 | 20 | 6851.40 (2542.54) | Reference |  |  |  |
| 2 | 20 | 6934.10 (2610.43) | 82.70 | -336.96 | 502.36 | 0.699 |
| 3 | 20 | 6913.75 (2597.11) | 62.35 | -357.31 | 482.01 | 0.771 |
| 5 | 20 | 6898.60 (2598.88) | 47.20 | -372.46 | 466.86 | 0.826 |
| 9 | 20 | 6902.95 (2613.47) | 51.55 | -368.11 | 471.21 | 0.810 |
| Layer |  |  |  |  |  |  |
| Superficial | 50 | 5085.30 (1443.96) | Reference |  |  |  |
| Deep | 50 | 8715.02 (2055.81) | 3629.72 | 3364.31 | 3895.14 | <0.001 |
| Sex |  |  |  |  |  |  |
| Female | 40 | 6042.50 (2070.01) | Reference |  |  |  |
| Gelding | 30 | 8523.43 (2358.65) | 2480.93 | 632.07 | 4329.80 | 0.009 |
| Entire | 30 | 6420.43 (2591.81) | 377.93 | -1470.93 | 2226.80 | 0.689 |
| Limb |  |  |  |  |  |  |
| Right | 70 | 7475.29 (2590.53) | Reference |  |  |  |
| Left | 30 | 5558.20 (1842.05) | -1917.09 | -3785.03 | -49.14 | 0.044 |
| Fracture |  |  |  |  |  |  |
| No | 50 | 7106.60 (2772.31) | Reference |  |  |  |
| Yes | 50 | 6693.72 (2293.60) | -412.88 | -2425.68 | 1599.92 | 0.688 |
| POD |  |  |  |  |  |  |
| 0 | 60 | 7393.63 (2334.11) | Reference |  |  |  |
| 1 | 40 | 6159.95 (2682.64) | -1233.68 | -3158.19 | 690.83 | 0.209 |
| Microcrack |  |  |  |  |  |  |
| 0 | 90 | 7084.81 (2572.04) | Reference |  |  |  |
| 1 | 10 | 5238.30 (1459.67) | -1846.51 | -5028.64 | 1335.62 | 0.255 |
| Resorption |  |  |  |  |  |  |
| 0 | 60 | 7891.15 (2291.68) | Reference |  |  |  |
| 1 | 40 | 5413.68 (2157.07) | -2477.48 | -3866.92 | -1088.03 | <0.001 |
| BVTV |  |  | 15822.30 | 10543.96 | 21100.64 | <0.001 |
| BMD |  |  | 38.89 | 21.41 | 56.36 | <0.001 |
| Age (months) |  |  | -173.13 | -359.30 | 13.04 | 0.068 |
| Cartilage (mm) |  |  | -12700.00 | -22700.00 | -2774.23 | 0.012 |
| Angle A |  |  | -237.32 | -800.94 | 326.30 | 0.409 |
| Angle B |  |  | -157.17 | -1233.37 | 919.04 | 0.775 |
| Even A |  |  | 1959.31 | -130.09 | 4048.71 | 0.066 |
| Even B |  |  | 2150.48 | -4022.64 | 8323.61 | 0.495 |
| DBVF |  |  | 19040.19 | 4361.56 | 33718.82 | 0.011 |
| DBV/BSA |  |  | 496000.00 | -47900.00 | 1040000.00 | 0.074 |

**Table 3.3.** Stiffness (MPa) of dorsal MCIII subchondral bone specimens with means, standard deviations (s.d.), and univariable mixed effects linear model estimated regression coefficients (Coef.), their 95% confidence intervals, and alpha level (P-value) of factors associated with stiffness (n = 100) of the subchondral bone at two depths (superficial 2 mm or deeper 2 mm) within each specimen from the metacarpophalangeal joint of n = 10 Thoroughbred racehorses. Adjusted for depth and cycle as fixed-effect categorical variables, and for horse-level random effects.

| **Variable** | **Number** | **Mean (s.d.)** | **Coef.** | **95% Confidence Interval** | | **P-value** |
| --- | --- | --- | --- | --- | --- | --- |
|  |  |  |  | **Lower**  **Bound** | **Upper**  **Bound** |  |
| Cycle |  |  |  |  |  |  |
| 1 | 20 | 5204.05 (1413.16) | Reference |  |  |  |
| 2 | 20 | 5106.70 (1466.15) | -97.35 | -313.57 | 118.87 | 0.378 |
| 3 | 20 | 5116.50 (1482.93) | -87.55 | -303.77 | 128.67 | 0.427 |
| 5 | 20 | 5129.35 (1489.05) | -74.70 | -290.92 | 141.52 | 0.498 |
| 9 | 20 | 5167.20 (1502.98) | -36.85 | -253.07 | 179.37 | 0.738 |
| Layer |  |  |  |  |  |  |
| Superficial | 50 | 4307.22 (1050.52) | Reference |  |  |  |
| Deep | 50 | 5982.30 (1289.86) | 1675.08 | 1538.33 | 1811.83 | <0.001 |
| Sex |  |  |  |  |  |  |
| Female | 40 | 5793.33 (1409.18) | Reference |  |  |  |
| Gelding | 30 | 5409.40 (1350.28) | -383.93 | -1612.19 | 844.34 | 0.540 |
| Entire | 30 | 4015.37 (792.94) | -1777.96 | -3006.22 | -549.70 | 0.005 |
| Limb |  |  |  |  |  |  |
| Right | 70 | 5155.57 (1330.23) | Reference |  |  |  |
| Left | 30 | 5119.53 (1698.10) | -36.04 | -1545.09 | 1473.01 | 0.963 |
| Fracture |  |  |  |  |  |  |
| No | 50 | 4991.52 (1445.71) | Reference |  |  |  |
| Yes | 50 | 5298.00 (1435.59) | 306.48 | -1063.64 | 1676.60 | 0.661 |
| POD |  |  |  |  |  |  |
| 0 | 60 | 5424.83 (1416.22) | Reference |  |  |  |
| 1 | 40 | 4724.65 (1392.71) | -700.18 | -2043.57 | 643.20 | 0.307 |
| Resorption |  |  |  |  |  |  |
| 0 | 80 | 5412.83 (1438.88) | Reference |  |  |  |
| 1 | 20 | 4072.50 (841.17) | -1340.33 | -2856.71 | 176.06 | 0.083 |
|  |  |  |  |  |  |  |
| BVTV |  |  | 4552.89 | 683.12 | 8422.66 | 0.021 |
| BMD |  |  | 34.19 | 21.48 | 46.91 | <0.001 |
| Age (months) |  |  | -87.46 | -223.55 | 48.64 | 0.208 |
| Cartilage (mm) |  |  | -9689.76 | -17400.00 | -1994.32 | 0.014 |
| Angle A |  |  | -99.75 | -515.96 | 316.47 | 0.639 |
| Angle B |  |  | -39.46 | -231.09 | 152.17 | 0.687 |
| Even A |  |  | 1687.34 | -209.58 | 3584.26 | 0.081 |
| Even B |  |  | -3294.42 | -7470.26 | 881.43 | 0.122 |
| DBVF |  |  | 37423.85 | 16948.93 | 57898.77 | <0.001 |
| DBV/BSA |  |  | 1800000.00 | -51900.00 | 3650000.00 | 0.057 |

**Table 3.4.** Stiffness (MPa) of proximal sesamoid subchondral bone specimens with means, standard deviations (s.d.), and univariable mixed effects linear model estimated regression coefficients (Coef.), their 95% confidence intervals, and alpha level (P-value) of factors associated with stiffness (n = 100) of the subchondral bone at two depths (superficial 2 mm or deeper 2 mm) within each specimen from the metacarpophalangeal joint of n = 10 Thoroughbred racehorses. Adjusted for depth and cycle as fixed-effect categorical variables, and for horse-level random effects.

| **Variable** | **Number** | **Mean (s.d.)** | **Coef.** | **95% Confidence Interval** | | **P-value** |
| --- | --- | --- | --- | --- | --- | --- |
|  |  |  |  | **Lower**  **Bound** | **Upper**  **Bound** |  |
| Cycle |  |  |  |  |  |  |
| 1 | 20 | 7089.40 (2688.03) | Reference |  |  |  |
| 2 | 20 | 7068.80 (2614.14) | -20.60 | -268.06 | 226.86 | 0.870 |
| 3 | 20 | 7054.70 (2546.94) | -34.70 | -282.16 | 212.76 | 0.783 |
| 5 | 20 | 7090.50 (2543.18) | 1.10 | -246.36 | 248.56 | 0.993 |
| 9 | 20 | 7148.30 (2520.49) | 58.90 | -188.56 | 306.36 | 0.641 |
| Layer |  |  |  |  |  |  |
| Superficial | 50 | 4933.52 (1226.61) | Reference |  |  |  |
| Deep | 50 | 9247.16 (1393.56) | 4313.64 | 4157.13 | 4470.15 | <0.001 |
| Sex |  |  |  |  |  |  |
| Female | 40 | 6703.88 (2624.14) | Reference |  |  |  |
| Gelding | 30 | 7921.83 (2505.15) | 1217.96 | -453.79 | 2889.71 | 0.153 |
| Entire | 30 | 6774.13 (2302.91) | 70.26 | -1601.49 | 1742.01 | 0.934 |
| Limb |  |  |  |  |  |  |
| Right | 70 | 7188.79 (2589.27) | Reference |  |  |  |
| Left | 30 | 6860.63 (2415.53) | -328.15 | -1996.58 | 1340.27 | 0.700 |
| Fracture |  |  |  |  |  |  |
| No | 50 | 7389.18 (2624.78) | Reference |  |  |  |
| Yes | 50 | 6791.50 (2422.50) | -597.68 | -2092.93 | 897.57 | 0.433 |
| POD |  |  |  |  |  |  |
| 0 | 60 | 7537.00 (2452.59) | Reference |  |  |  |
| 1 | 40 | 6420.35 (2528.04) | -1116.65 | -2528.35 | 295.05 | 0.121 |
| Resorption |  |  |  |  |  |  |
| 0 | 70 | 7090.36 (2549.42) | Reference |  |  |  |
| 1 | 30 | 7090.30 (2529.82) | -0.06 | -1680.83 | 1680.72 | 1.000 |
|  |  |  |  |  |  |  |
| BVTV |  |  | 4887.82 | 80.68 | 9694.95 | 0.046 |
| BMD |  |  | -3.39 | -14.90 | 8.13 | 0.564 |
| Age (months) |  |  | -115.70 | -262.24 | 30.83 | 0.122 |
| Cartilage (mm) |  |  | -4870.37 | -12100.00 | 2378.52 | 0.188 |
| Angle A |  |  | 265.05 | -410.55 | 940.64 | 0.442 |
| Angle B |  |  | -379.67 | -759.02 | -.32356 | 0.050 |
| Even A |  |  | 1457.42 | -3286.98 | 6201.82 | 0.547 |
| Even B |  |  | 1919.00 | -3307.57 | 7145.57 | 0.472 |
| DBVF |  |  | 14117.04 | -259.66 | 28493.74 | 0.054 |
| DBV/BSA |  |  | 241000.00 | -203000.00 | 685000.00 | 0.288 |

**Table 3.5.** Multivariable mixed effects linear model estimated regression coefficients (Coef.), their 95% confidence intervals, and alpha level (P-value) of factors associated with stiffness of palmar MCIII subchondral bone at two depths (superficial 2 mm or deeper 2 mm) within each specimen from the metacarpophalangeal joint of n = 10 Thoroughbred racehorses. The number of observations for each variable is 100.

| **Variable** | **Coef.** | **95% Confidence Interval** | | **P-value** |
| --- | --- | --- | --- | --- |
|  |  | **Lower Bound** | **Upper Bound** |  |
| Layer |  |  |  |  |
| Superficial | Reference |  |  |  |
| Deep | 3039.80 | 2503.40 | 3576.20 | <0.001 |
| BVTV | 26931.53 | 23188.62 | 30674.44 | <0.001 |
| BMD | -809.44 | -929.60 | -689.27 | <0.001 |
| BMD^2^ | 0.47 | 0.40 | 0.54 | <0.001 |
| Cycle |  |  |  |  |
| 1 | Reference |  |  |  |
| 2 | 82.70 | -121.92 | 287.32 | 0.428 |
| 3 | 62.35 | -142.27 | 266.97 | 0.550 |
| 5 | 47.20 | -157.42 | 251.82 | 0.651 |
| 9 | 51.55 | -153.07 | 256.17 | 0.621 |
| Constant | 327000.00 | 275000.00 | 379000.00 | <0.001 |
| Constant | 7.48 | 7.02 | 7.98 |  |
| Constant | 5.80 | 5.65 | 5.95 |  |

**Table 3.6.** Multivariable mixed effects linear model estimated regression coefficients (Coef.), their 95% confidence intervals, and alpha level (P-value) of factors associated with stiffness of dorsal MCIII subchondral bone at two depths (superficial 2 mm or deeper 2 mm) within each specimen from the metacarpophalangeal joint of n = 10 Thoroughbred racehorses. The number of observations for each variable is 95.

| **Variable** | **Coef.** | **95% Confidence Interval** | | **P-value** |
| --- | --- | --- | --- | --- |
|  |  | **Lower Bound** | **Upper Bound** |  |
| Layer |  |  |  |  |
| Superficial | Reference |  |  |  |
| Deep | 1447.87 | 1018.01 | 1877.73 | <0.001 |
| DBV/BSA | 1690000.00 | -2870000.00 | -514000.00 | 0.005 |
| Layer # DBV/BSA interaction |  |  |  |  |
| Superficial # DBV/BSA | Reference |  |  |  |
| Deep # DBV/BSA | -15900000.00 | -18500000.00 | -13200000.00 | <0.001 |
| BMD | 33.69 | 25.10 | 42.27 | <0.001 |
| Cycle |  |  |  |  |
| 1 | Reference |  |  |  |
| 2 | -103.79 | -223.28 | 15.70 | 0.089 |
| 3 | -93.68 | -213.17 | 25.80 | 0.124 |
| 5 | -80.26 | -199.75 | 39.22 | 0.188 |
| 9 | -39.79 | -159.28 | 79.70 | 0.514 |
| Constant | -24100.00 | -31500.00 | -16800.00 | <0.001 |
| Constant | 6.57 | 6.14 | 7.02 |  |
| Constant | 5.24 | 5.09 | 5.39 |  |

**Table 3.7.** Multivariable mixed effects linear model estimated regression coefficients (Coef.), their 95% confidence intervals, and alpha level (P-value) of factors associated with stiffness of proximal sesamoid subchondral bone at two depths (superficial 2 mm or deeper 2 mm) within each specimen from the metacarpophalangeal joint of n = 10 Thoroughbred racehorses. The number of observations for each variable is 100.

| **Variable** | **Coef.** | **95% Confidence Interval** | | **P-value** |
| --- | --- | --- | --- | --- |
|  |  | **Lower Bound** | **Upper Bound** |  |
| Layer |  |  |  |  |
| Superficial | Reference |  |  |  |
| Deep | 1843.37 | 1190.72 | 2496.03 | <0.001 |
| Cartilage (mm) | -1825.19 | -6236.09 | 2585.72 | 0.417 |
| Layer # Cartilage interaction |  |  |  |  |
| Superficial # Cartilage | Reference |  |  |  |
| Deep # Cartilage | 4911.90 | 3815.21 | 6008.59 | <0.001 |
| DBV/BSA | 3990000.00 | 2930000.00 | 5060000.00 | <0.001 |
| DBV/BSA^2^ | -1840000000.00 | -2430000000.00 | -1250000000.00 | <0.001 |
| Sex |  |  |  |  |
| Female | Reference |  |  |  |
| Gelding | 189.78 | -845.82 | 1225.38 | 0.719 |
| Male Entire | -1251.56 | -2437.74 | -65.37 | 0.039 |
| Age (months) | 4645.15 | 2461.26 | 6829.05 | <0.001 |
| Age^2^ | -50.83 | -74.41 | -27.25 | <0.001 |
| Cycle |  |  |  |  |
| 1 | Reference |  |  |  |
| 2 | -20.60 | -175.67 | 134.47 | 0.795 |
| 3 | -34.70 | -189.77 | 120.37 | 0.661 |
| 5 | 1.10 | -153.97 | 156.17 | 0.989 |
| 9 | 58.90 | -96.17 | 213.97 | 0.457 |
| Constant | -100000.00 | -151000.00 | -49500.00 | <0.001 |
| Constant | 6.26 | 5.83 | 6.73 |  |
| Constant | 5.52 | 5.38 | 5.67 |  |

| 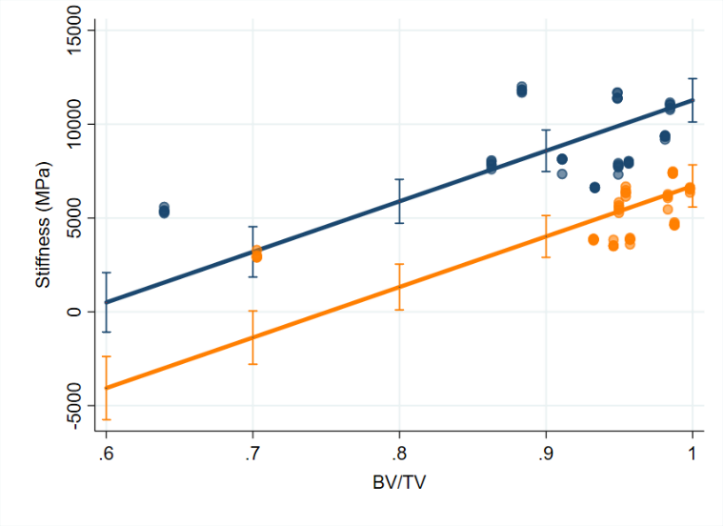   1. Palmar MCIII | 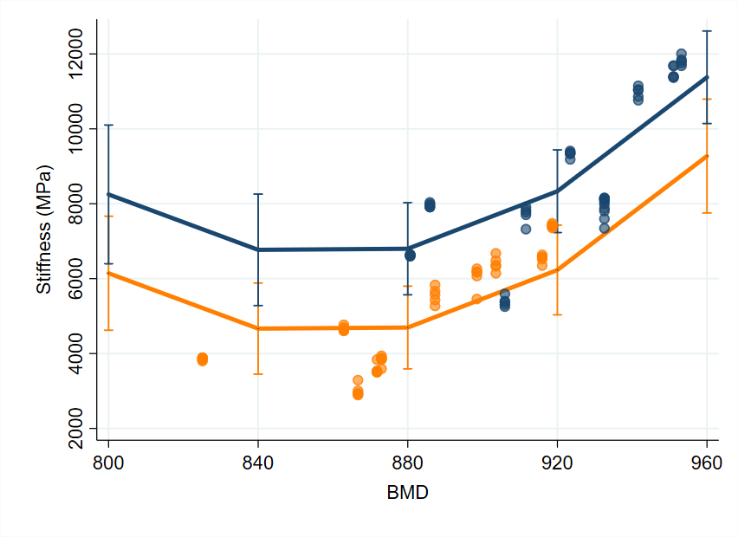   1. Palmar MCIII |
| --- | --- |
| 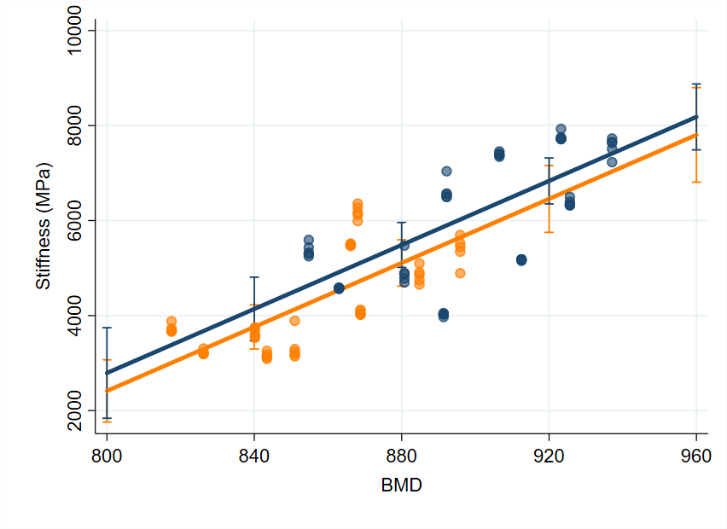   1. Dorsal MCIII | 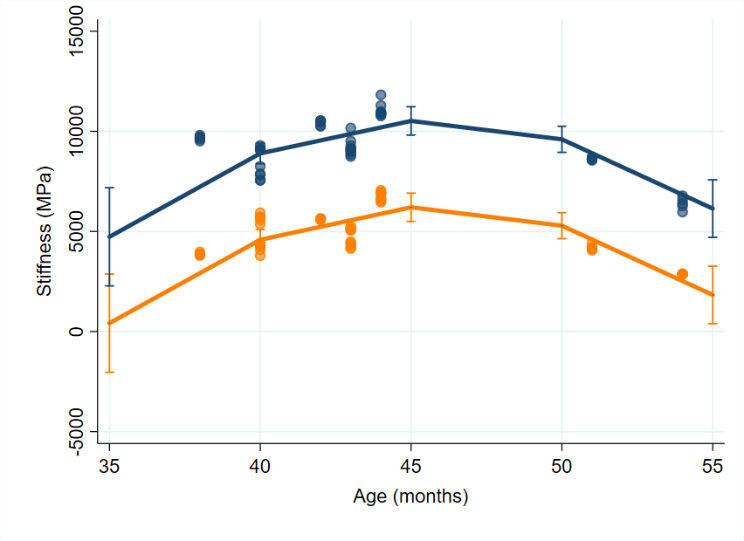   1. Sesamoid |
| 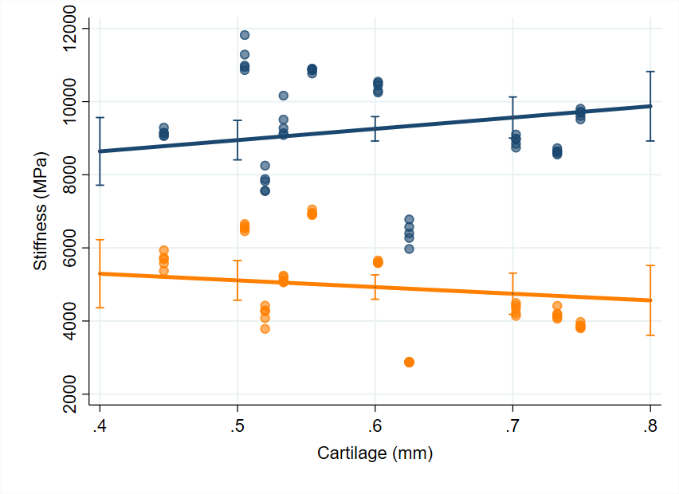   1. Sesamoid |  |

**Figure 3.1.** Adjusted margins plots of the relationship between study factors and stiffness (MPa) of subchondral bone in Thoroughbred racehorses (n = 10) with 95% confidence intervals. The deeper 2 mm subchondral bone layer is depicted in navy, and superficial 2 mm layer in orange. **(A)** Associations between bone volume fraction (BVTV) and stiffness at the palmar MCIII site. **(B)** Associations between bone mineral density (BMD) and stiffness at the palmar MCIII site. **(C)** Associations between bone mineral density (BMD) and stiffness at the dorsal MCIII site. **(D)** Associations between horse age (months) and stiffness at the proximal sesamoid site. **(E)** Associations between cartilage thickness (mm) and stiffness at the proximal sesamoid site.

| 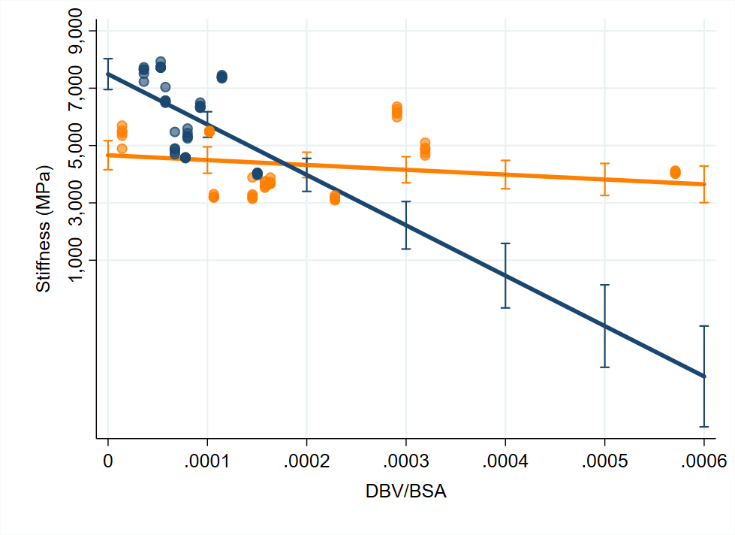   1. Dorsal MCIII | 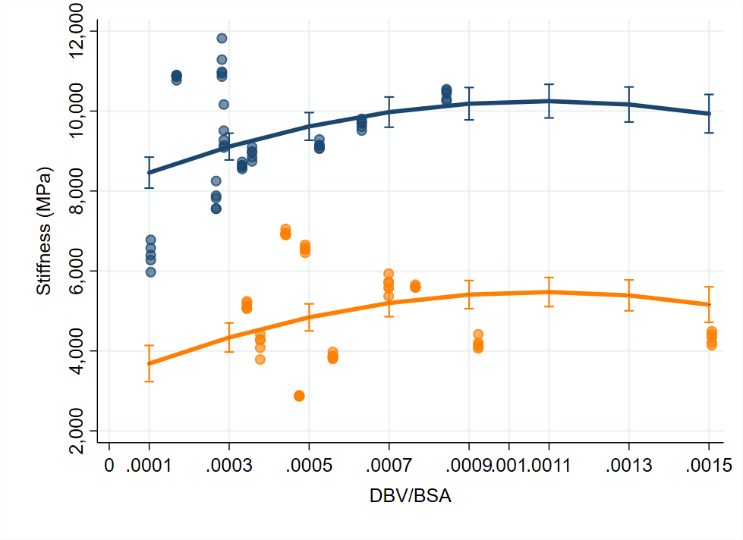   1. Sesamoid |
| --- | --- |

**Figure 3.2.** Adjusted margins plots of the relationship between adjusted damaged bone volume fraction (DBV/BSA, in mm^-2^) and stiffness (MPa) of subchondral bone in Thoroughbred racehorses (n = 10 except for the deep dorsal layer where n = 9) with 95% confidence intervals. The deeper 2 mm of subchondral bone layer is depicted in navy, and superficial 2 mm layer in orange. **(A)** Associations at the dorsal MCIII site. **(B)** Associations at the proximal sesamoid site.
