# Supplementary material for: Biomechanical and Microstructural Properties of Subchondral Bone From Three Metacarpophalangeal Joint Sites in Thoroughbred Racehorses
Source: Front Vet Sci. 2022 Jun 28;9:923356. doi: 10.3389/fvets.2022.923356 (PMC9277662; doi:10.3389/fvets.2022.923356)
Supplement: Supplementary file 1 [file Data_Sheet_1.zip › Supplementary Item 4.docx]

Supplementary Item 4. Associations between hysteresis and study factors.

**Table 4.1.** Normalized hysteresis (fraction of energy loss) of total cartilage-bone specimen with means, standard deviations (s.d.), and univariable mixed effects linear model estimated regression coefficients (Coef.), their 95% confidence intervals, and alpha level (P-value) of factors associated with total specimen hysteresis (n = 420) of three sites (dorsal MCIII, palmar MCIII, or proximal sesamoid) within the metacarpophalangeal joint of n = 10 Thoroughbred racehorses. Adjusted for multiple measurements of site and cycle within horse to account for horse-level random effects.

| **Variable** | **Number** | **Mean (s.d.)** | **Coef.** | **95% Confidence Interval** | | **P-value** |
| --- | --- | --- | --- | --- | --- | --- |
|  |  |  |  | **Lower**  **Bound** | **Upper**  **Bound** |  |
| Cycle |  |  |  |  |  |  |
| 1 | 30 | 0.30 (0.07) | Reference |  |  |  |
| 2 | 30 | 0.19 (0.04) | -0.11 | -0.12 | -0.09 | <0.001 |
| 3 | 30 | 0.18 (0.03) | -0.12 | -0.14 | -0.11 | <0.001 |
| 5 | 30 | 0.16 (0.03) | -0.14 | -0.15 | -0.12 | <0.001 |
| 9 | 30 | 0.15 (0.03) | -0.15 | -0.16 | -0.13 | <0.001 |
| 19 | 30 | 0.14 (0.03) | -0.16 | -0.17 | -0.14 | <0.001 |
| 29 | 30 | 0.14 (0.03) | -0.16 | -0.18 | -0.15 | <0.001 |
| 39 | 30 | 0.13 (0.03) | -0.17 | -0.18 | -0.15 | <0.001 |
| 49 | 30 | 0.13 (0.03) | -0.17 | -0.18 | -0.15 | <0.001 |
| 59 | 30 | 0.13 (0.03) | -0.17 | -0.18 | -0.16 | <0.001 |
| 69 | 30 | 0.13 (0.03) | -0.17 | -0.18 | -0.16 | <0.001 |
| 79 | 30 | 0.13 (0.03) | -0.17 | -0.19 | -0.16 | <0.001 |
| 89 | 30 | 0.13 (0.03) | -0.17 | -0.19 | -0.16 | <0.001 |
| 99 | 30 | 0.12 (0.03) | -0.17 | -0.19 | -0.16 | <0.001 |
| Site |  |  |  |  |  |  |
| Sesamoid | 140 | 0.16 (0.06) | Reference |  |  |  |
| Dorsal | 140 | 0.14 (0.04) | -0.02 | -0.03 | -0.007 | 0.002 |
| Palmar | 140 | 0.16 (0.05) | -0.001 | -0.01 | 0.01 | 0.828 |
| Sex |  |  |  |  |  |  |
| Entire | 126 | 0.16 (0.05) | Reference |  |  |  |
| Female | 168 | 0.16 (0.06) | 0.01 | -0.04 | 0.01 | 0.275 |
| Gelding | 126 | 0.15 (0.06) | 0.004 | -0.03 | 0.02 | 0.780 |
| Limb |  |  |  |  |  |  |
| Right | 294 | 0.15 (0.05) | Reference |  |  |  |
| Left | 126 | 0.16 (0.06) | 0.01 | -0.01 | 0.03 | 0.393 |
| Fracture |  |  |  |  |  |  |
| Yes | 210 | 0.15 (0.05) | Reference |  |  |  |
| No | 210 | 0.16 (0.06) | 0.009 | -0.03 | 0.01 | 0.453 |
| POD |  |  |  |  |  |  |
| 0 | 252 | 0.15 (0.06) | Reference |  |  |  |
| 1 | 168 | 0.16 (0.05) | 0.10 | -0.008 | 0.04 | 0.219 |
| Microcrack |  |  |  |  |  |  |
| 0 | 406 | 0.15 (0.05) | Reference |  |  |  |
| 1 | 14 | 0.22 (0.04) | 0.062 | 0.03 | 0.09 | <0.001 |
| Resorption |  |  |  |  |  |  |
| 0 | 294 | 0.15 (0.05) | Reference |  |  |  |
| 1 | 126 | 0.17 (0.06) | 0.02 | 0.006 | 0.03 | 0.005 |
|  |  |  |  |  |  |  |
| BVTV |  |  | 0.08 | 0.02 | 0.14 | 0.007 |
| BMD |  |  | 0.0001 | -0.0001 | 0.0003 | 0.310 |
| Age (months) |  |  | 0.002 | -0.0004 | 0.004 | 0.118 |
| Cartilage (mm) |  |  | 0.11 | 0.04 | 0.19 | 0.001 |
| Angle A |  |  | 0.004 | 0.0005 | 0.007 | 0.026 |
| Angle B |  |  | 0.0009 | -0.001 | 0.003 | 0.455 |
| Even A |  |  | -0.007 | -0.01 | -0.002 | 0.010 |
| Even B |  |  | -0.02 | -0.04 | -0.01 | <0.001 |

**Table 4.2.** Normalized hysteresis (fraction of energy loss) of palmar MCIII subchondral bone specimens with means, standard deviations (s.d.), and univariable mixed effects linear model estimated regression coefficients (Coef.), their 95% confidence intervals, and alpha level (P-value) of factors associated with hysteresis (n = 100) of the subchondral bone at two depths (superficial 2 mm or deeper 2 mm) within each specimen from the metacarpophalangeal joint of n = 10 Thoroughbred racehorses. Adjusted for depth and cycle as fixed-effect categorical variables, and for horse-level random effects.

| **Variable** | **Number** | **Mean (s.d.)** | **Coef.** | **95% Confidence Interval** | | **P-value** |
| --- | --- | --- | --- | --- | --- | --- |
|  |  |  |  | **Lower**  **Bound** | **Upper**  **Bound** |  |
| Cycle |  |  |  |  |  |  |
| 1 | 20 |  | Reference |  |  |  |
| 2 | 20 |  | -0.04 | -0.06 | -0.02 | <0.001 |
| 3 | 20 |  | -0.05 | -0.07 | -0.03 | <0.001 |
| 5 | 20 |  | -0.05 | -0.07 | -0.03 | <0.001 |
| 9 | 20 |  | -0.06 | -0.08 | -0.03 | <0.001 |
| Layer |  |  |  |  |  |  |
| Superficial | 50 |  | Reference |  |  |  |
| Deep | 50 |  | -0.15 | -0.16 | -0.13 | <0.001 |
| Sex |  |  |  |  |  |  |
| Female | 40 |  | Reference |  |  |  |
| Gelding | 30 |  | -0.05 | -0.11 | 0.006 | 0.077 |
| Entire | 30 |  | 0.01 | -0.05 | 0.07 | 0.659 |
| Limb |  |  |  |  |  |  |
| Right | 70 |  | Reference |  |  |  |
| Left | 30 |  | -0.02 | -0.08 | 0.05 | 0.565 |
| Fracture |  |  |  |  |  |  |
| No | 50 |  | Reference |  |  |  |
| Yes | 50 |  | 0.04 | -0.02 | 0.09 | 0.197 |
| POD |  |  |  |  |  |  |
| 0 | 60 |  | Reference |  |  |  |
| 1 | 40 |  | 0.07 | 0.03 | 0.11 | 0.001 |
| Microcrack |  |  |  |  |  |  |
| 0 | 90 |  | Reference |  |  |  |
| 1 | 10 |  | 0.05 | -0.05 | 0.14 | 0.333 |
| Resorption |  |  |  |  |  |  |
| 0 | 60 |  | Reference |  |  |  |
| 1 | 40 |  | 0.06 | 0.02 | 0.11 | 0.009 |
|  |  |  |  |  |  |  |
| BVTV |  |  | -0.58 | -0.85 | -0.31 | <0.001 |
| BMD |  |  | -0.002 | -0.003 | -0.001 | <0.001 |
| Age (months) |  |  | 0.004 | -0.002 | 0.01 | 0.163 |
| Cartilage (mm) |  |  | 0.51 | 0.32 | 0.70 | <0.001 |
| Angle A |  |  | 0.01 | -0.004 | 0.03 | 0.159 |
| Angle B |  |  | -0.00003 | -0.03 | 0.03 | 0.998 |
| Even A |  |  | -0.05 | -0.11 | 0.01 | 0.121 |
| Even B |  |  | -0.11 | -0.28 | 0.06 | 0.197 |
| DBVF |  |  | 0.38 | -0.38 | 1.15 | 0.327 |
| DBV/BSA |  |  | -12.45 | -40.27 | 15.37 | 0.380 |

**Table 4.3.** Normalized hysteresis (fraction of energy loss) of dorsal MCIII subchondral bone specimens with means, standard deviations (s.d.), and univariable mixed effects linear model estimated regression coefficients (Coef.), their 95% confidence intervals, and alpha level (P-value) of factors associated with hysteresis (n = 100) of the subchondral bone at two depths (superficial 2 mm or deeper 2 mm) within each specimen from the metacarpophalangeal joint of n = 10 Thoroughbred racehorses. Adjusted for depth and cycle as fixed-effect categorical variables, and for horse-level random effects.

| **Variable** | **Number** | **Mean (s.d.)** | **Coef.** | **95% Confidence Interval** | | **P-value** |
| --- | --- | --- | --- | --- | --- | --- |
|  |  |  |  | **Lower**  **Bound** | **Upper**  **Bound** |  |
| Cycle |  |  |  |  |  |  |
| 1 | 20 |  | Reference |  |  |  |
| 2 | 20 |  | -0.06 | -0.09 | -0.04 | <0.001 |
| 3 | 20 |  | -0.07 | -0.10 | -0.05 | <0.001 |
| 5 | 20 |  | -0.08 | -0.10 | -0.06 | <0.001 |
| 9 | 20 |  | -0.08 | -0.11 | -0.06 | <0.001 |
| Layer |  |  |  |  |  |  |
| Superficial | 50 |  | Reference |  |  |  |
| Deep | 50 |  | -0.17 | -0.19 | -0.15 | <0.001 |
| Sex |  |  |  |  |  |  |
| Female | 40 |  | Reference |  |  |  |
| Gelding | 30 |  | 0.05 | -0.04 | 0.13 | 0.320 |
| Entire | 30 |  | -0.03 | -0.12 | 0.06 | 0.545 |
| Limb |  |  |  |  |  |  |
| Right | 70 |  | Reference |  |  |  |
| Left | 30 |  | -0.02 | -0.11 | 0.07 | 0.705 |
| Fracture |  |  |  |  |  |  |
| No | 50 |  | Reference |  |  |  |
| Yes | 50 |  | -0.04 | -0.12 | 0.04 | 0.335 |
| POD |  |  |  |  |  |  |
| 0 | 60 |  | Reference |  |  |  |
| 1 | 40 |  | -0.02 | -0.11 | 0.06 | 0.593 |
| Resorption |  |  |  |  |  |  |
| 0 | 60 |  | Reference |  |  |  |
| 1 | 40 |  | -0.07 | -0.16 | 0.02 | 0.144 |
|  |  |  |  |  |  |  |
| BVTV |  |  | 0.01 | -0.29 | 0.32 | 0.930 |
| BMD |  |  | -0.001 | -0.002 | 0.0005 | 0.257 |
| Age (months) |  |  | -0.004 | -0.01 | 0.005 | 0.390 |
| Cartilage (mm) |  |  | -0.14 | -0.71 | 0.43 | 0.625 |
| Angle A |  |  | -0.006 | -0.03 | 0.02 | 0.629 |
| Angle B |  |  | -0.01 | -0.02 | 0.0001 | 0.053 |
| Even A |  |  | -0.02 | -0.15 | 0.11 | 0.758 |
| Even B |  |  | -0.16 | -0.42 | 0.09 | 0.207 |
| DBVF |  |  | 1.37 | -0.35 | 3.09 | 0.118 |
| DBV/BSA |  |  | -94.17 | -240.24 | 51.90 | 0.206 |

**Table 4.4.** Normalized hysteresis (fraction of energy loss) of proximal sesamoid subchondral bone specimens with means, standard deviations (s.d.), and univariable mixed effects linear model estimated regression coefficients (Coef.), their 95% confidence intervals, and alpha level (P-value) of factors associated with hysteresis (n = 100) of the subchondral bone at two depths (superficial 2 mm or deeper 2 mm) within each specimen from the metacarpophalangeal joint of n = 10 Thoroughbred racehorses Adjusted for depth and cycle as fixed-effect categorical variables, and for horse-level random effects.

| **Variable** | **Number** | **Mean (s.d.)** | **Coef.** | **95% Confidence Interval** | | **P-value** |
| --- | --- | --- | --- | --- | --- | --- |
|  |  |  |  | **Lower**  **Bound** | **Upper**  **Bound** |  |
| Cycle |  |  |  |  |  |  |
| 1 | 20 |  | Reference |  |  |  |
| 2 | 20 |  | -0.09 | -0.12 | -0.06 | <0.001 |
| 3 | 20 |  | -0.11 | -0.13 | -0.08 | <0.001 |
| 5 | 20 |  | -0.12 | -0.15 | -0.09 | <0.001 |
| 9 | 20 |  | -0.13 | -0.16 | -0.10 | <0.001 |
| Layer |  |  |  |  |  |  |
| Superficial | 50 |  | Reference |  |  |  |
| Deep | 50 |  | -0.26 | -0.29 | -0.23 | <0.001 |
| Sex |  |  |  |  |  |  |
| Female | 40 |  | Reference |  |  |  |
| Gelding | 30 |  | -0.12 | -0.19 | -0.05 | 0.001 |
| Entire | 30 |  | -0.03 | -0.102 | 0.04 | 0.364 |
| Limb |  |  |  |  |  |  |
| Right | 70 |  | Reference |  |  |  |
| Left | 30 |  | 0.02 | -0.07 | 0.11 | 0.649 |
| Fracture |  |  |  |  |  |  |
| No | 50 |  | Reference |  |  |  |
| Yes | 50 |  | 0.09 | 0.03 | 0.15 | 0.005 |
| **POD** |  |  |  |  |  |  |
| **0** | **60** |  | **Reference** |  |  |  |
| **1** | **40** |  |  |  |  |  |
| Resorption |  |  |  |  |  |  |
| 0 | 60 |  | Reference |  |  |  |
| 1 | 40 |  | -0.07 | -0.15 | 0.02 | 0.126 |
|  |  |  |  |  |  |  |
| BVTV |  |  | 0.37 | -0.07 | 0.82 | 0.101 |
| BMD |  |  | 0.0004 | -0.001 | 0.001 | 0.498 |
| Age (months) |  |  | 0.007 | -0.001 | 0.02 | 0.106 |
| Cartilage (mm) |  |  | 0.13 | -0.30 | 0.55 | 0.554 |
| Angle A |  |  | -0.01 | -0.05 | 0.03 | 0.567 |
| Angle B |  |  | 0.02 | 0.004 | 0.04 | 0.020 |
| Even A |  |  | 0.16 | -0.09 | 0.41 | 0.207 |
| Even B |  |  | 0.12 | -0.16 | 0.41 | 0.396 |
| DBVF |  |  | 0.92 | -0.44 | 2.29 | 0.186 |
| DBV/BSA |  |  | 47.36 | 5.20 | 89.52 | 0.028 |

**Table 4.5.** Multivariable mixed effects linear model estimated regression coefficients (Coef.), their 95% confidence intervals, and alpha level (P-value) of factors associated with normalized hysteresis (fraction of energy loss) of palmar MCIII subchondral bone at two depths (superficial 2 mm or deeper 2 mm) within each specimen from the metacarpophalangeal joint of n = 10 Thoroughbred racehorses. The number of observations for each variable is 100.

| **Variable** | **Coef.** | **95% Confidence**  **Interval** | | **P-value** |
| --- | --- | --- | --- | --- |
|  |  | **Lower Bound** | **Upper Bound** |  |
| Layer |  |  |  |  |
| Superficial | Reference |  |  |  |
| Deep | -0.17 | -0.20 | -0.15 | <0.001 |
| Cycle |  |  |  |  |
| 1 | Reference |  |  |  |
| 2 | -0.06 | -0.08 | -0.04 | <0.001 |
| 3 | -0.07 | -0.09 | -0.05 | <0.001 |
| 5 | -0.08 | -0.10 | -0.06 | <0.001 |
| 9 | -0.08 | -0.10 | -0.06 | <0.001 |
| Layer # Cycle interaction |  |  |  |  |
| Superficial # Cycle | Reference |  |  |  |
| Deep # Cycle 1 | 0.00 |  |  |  |
| Deep # Cycle 2 | 0.04 | 0.01 | 0.07 | 0.005 |
| Deep # Cycle 3 | 0.04 | 0.02 | 0.07 | 0.002 |
| Deep # Cycle 5 | 0.05 | 0.02 | 0.08 | 0.001 |
| Deep # Cycle 9 | 0.05 | 0.02 | 0.08 | <0.001 |
| Fracture |  |  |  |  |
| No | Reference |  |  |  |
| Yes | 0.07 | 0.05 | 0.10 | <0.001 |
| Limb |  |  |  |  |
| Right | Reference |  |  |  |
| Left | -0.09 | -0.12 | -0.06 | <0.001 |
| BVTV | 2.47 | 1.20 | 3.74 | <0.001 |
| BVTV^2^ | -1.70 | -2.43 | -0.97 | <0.001 |
| BMD | -0.02 | -0.03 | -0.01 | <0.001 |
| BMD^2^ | 0.00001 | 6.41e^-06^ | 0.00001 | <0.001 |
| Cartilage thickness | 0.54 | 0.43 | 0.65 | <0.001 |
| Even A | 0.11 | 0.06 | 0.17 | <0.001 |
| Constant | 8.36 | 4.92 | 11.80 | <0.001 |
| Constant | -24.86 |  |  |  |
| Constant | -3.80 |  |  |  |

**Table 4.6.** Multivariable mixed effects linear model estimated regression coefficients (Coef.), their 95% confidence intervals, and alpha level (P-value) of factors associated with normalized hysteresis (fraction of energy loss) of dorsal MCIII subchondral bone at two depths (superficial 2 mm or deeper 2 mm) within each specimen from the metacarpophalangeal joint of n = 10 Thoroughbred racehorses. The number of observations for each variable is 100.

| **Variable** | **Coef.** | **95% Confidence**  **Interval** | | **P-value** |
| --- | --- | --- | --- | --- |
|  |  | **Lower Bound** | **Upper Bound** |  |
| Layer |  |  |  |  |
| Superficial | Reference |  |  |  |
| Deep | -0.04 | -0.10 | 0.01 | 0.118 |
| Cycle |  |  |  |  |
| 1 | Reference |  |  |  |
| 2 | -0.06 | -0.08 | -0.05 | <0.001 |
| 3 | -0.07 | -0.09 | -0.06 | <0.001 |
| 5 | -0.08 | -0.10 | -0.06 | <0.001 |
| 9 | -0.08 | -0.10 | -0.07 | <0.001 |
| Layer # Cycle interaction |  |  |  |  |
| Superficial # Cycle | Reference |  |  |  |
| Deep # Cycle 1 | 0.00 |  |  |  |
| Deep # Cycle 2 | 0.05 | 0.03 | 0.07 | <0.001 |
| Deep # Cycle 3 | 0.06 | 0.03 | 0.08 | <0.001 |
| Deep # Cycle 5 | 0.06 | 0.04 | 0.08 | <0.001 |
| Deep # Cycle 9 | 0.06 | 0.04 | 0.09 | <0.001 |
| BVTV | 1.89 | 0.79 | 2.98 | 0.001 |
| BVTV^2^ | -1.00 | -1.64 | -0.36 | 0.002 |
| BMD | -0.01 | -0.03 | -0.003 | 0.014 |
| BMD^2^ | 7.05e^-06^ | 7.61e^-07^ | 0.00001 | 0.028 |
| Constant | 6.40 | 1.03 | 11.78 | 0.019 |
| Constant | -2.51 |  |  |  |
| Constant | -3.98 |  |  |  |

**Table 4.7.** Multivariable mixed effects linear model estimated regression coefficients (Coef.), their 95% confidence intervals, and alpha level (P-value) of factors associated with normalized hysteresis (fraction of energy loss) of proximal sesamoid subchondral bone at two depths (superficial 2 mm or deeper 2 mm) within each specimen from the metacarpophalangeal joint of n = 10 Thoroughbred racehorses. The number of observations for each variable is 100.

| **Variable** | **Coef.** | **95% Confidence**  **Interval** | | **P-value** |
| --- | --- | --- | --- | --- |
|  |  | **Lower Bound** | **Upper Bound** |  |
| Layer |  |  |  |  |
| Superficial | Reference |  |  |  |
| Deep | 0.60 | 0.307 | 0.899 | <0.001 |
| Cycle |  |  |  |  |
| 1 | Reference |  |  |  |
| 2 | -0.09 | -0.12 | -0.07 | <0.001 |
| 3 | -0.11 | -0.13 | -0.08 | <0.001 |
| 5 | -0.12 | -0.14 | -0.10 | <0.001 |
| 9 | -0.13 | -0.15 | -0.11 | <0.001 |
| Layer # Cycle interaction |  |  |  |  |
| Superficial # Cycle | Reference |  |  |  |
| Deep # Cycle 1 | 0.00 |  |  |  |
| Deep # Cycle 2 | 0.07 | 0.04 | 0.11 | <0.001 |
| Deep # Cycle 3 | 0.09 | 0.06 | 0.12 | <0.001 |
| Deep # Cycle 5 | 0.10 | 0.07 | 0.13 | <0.001 |
| Deep # Cycle 9 | 0.11 | 0.07 | 0.14 | <0.001 |
| BVTV | 1.578 | 1.087 | 2.070 | <0.001 |
| Layer # BVTV interaction |  |  |  |  |
| Superficial # BVTV | Reference |  |  |  |
| Deep # BVTV | -0.841 | -1.140 | -0.543 | <0.001 |
| BMD | -0.040 | -0.059 | -0.020 | <0.001 |
| BMD^2^ | 0.00002 | 0.00001 | 0.00003 | <0.001 |
| Age (months) | 0.009 | 0.001 | 0.018 | 0.029 |
| Constant | 17.127 | 8.168 | 26.085 | <0.001 |
| Constant | -2.775 |  |  |  |
| Constant | -3.631 |  |  |  |

| 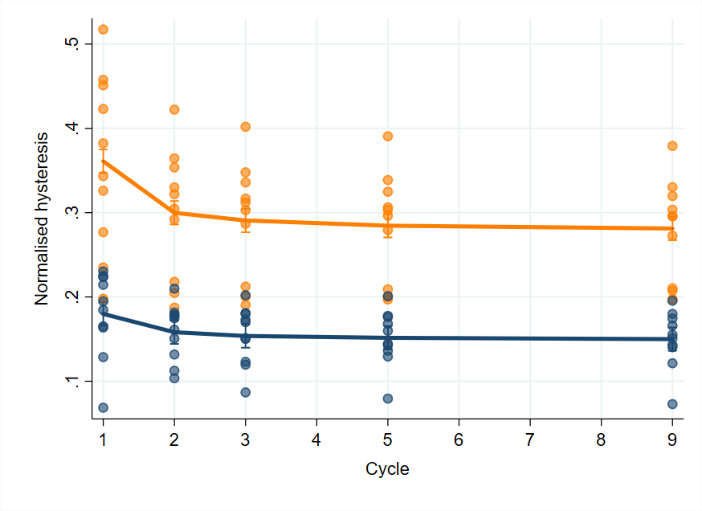   1. Palmar MCIII | 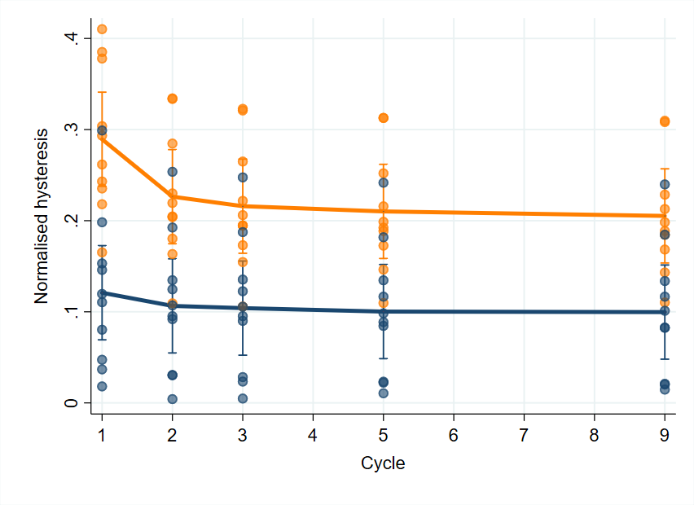   1. Dorsal MCIII |
| --- | --- |
| 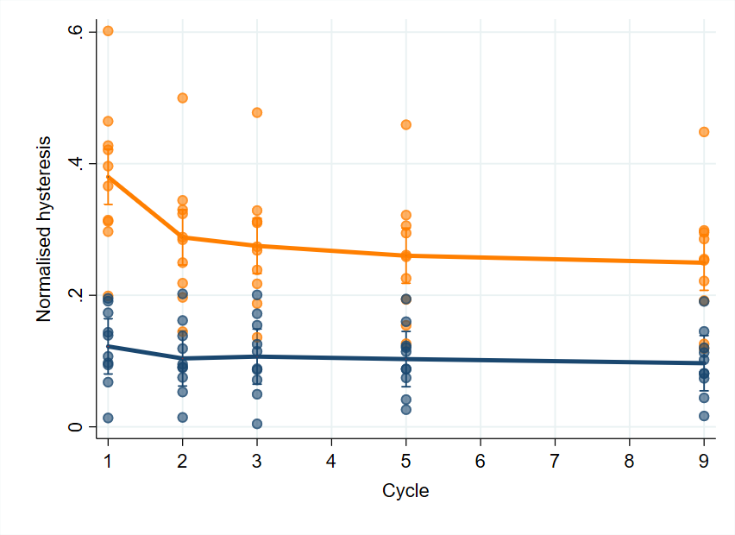   1. Sesamoid |  |

**Figure 4.1.** Adjusted margins plots of the relationship between cycle and normalized hysteresis (fraction of energy loss) of subchondral bone in Thoroughbred racehorses (n = 10) with 95% confidence intervals. The deeper 2 mm subchondral bone layer is depicted in navy, and superficial 2 mm layer in orange. **(A)** Associations at the palmar MCIII site. **(B)** Associations at the dorsal MCIII site. **(C)** Associations at the proximal sesamoid site.

| 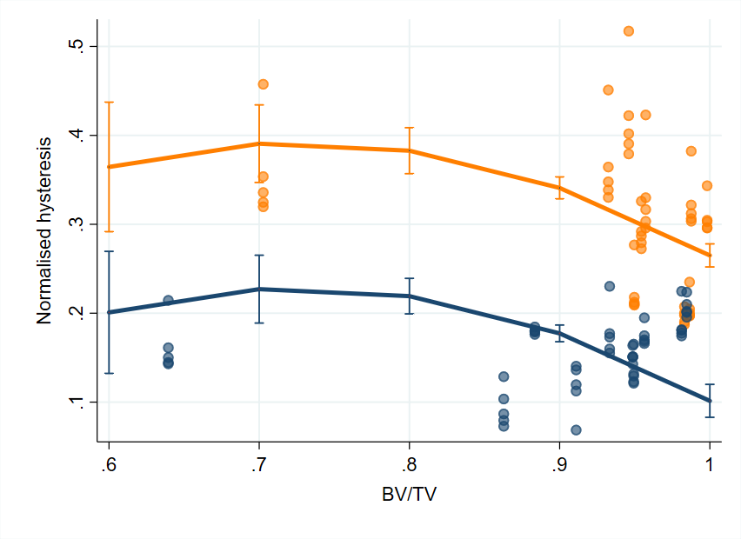   1. Palmar MCIII | 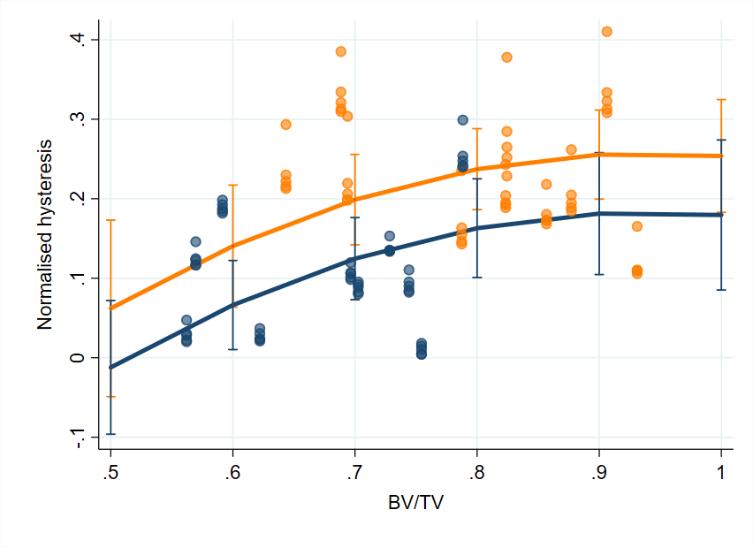   1. Dorsal MCIII |
| --- | --- |
| 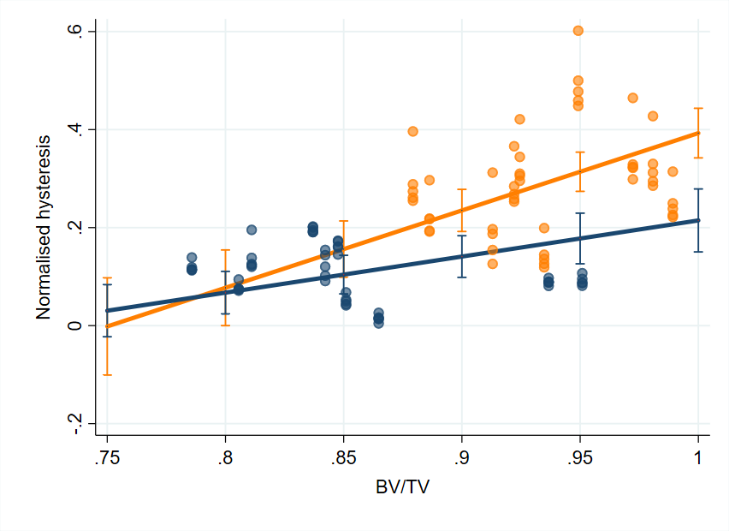   1. Sesamoid |  |

**Figure 4.2.** Adjusted margins plots of the relationship between normalized hysteresis (fraction of energy loss) and bone volume fraction (BVTV) of subchondral bone in Thoroughbred racehorses (n = 10) with 95% confidence intervals. The deeper 2 mm subchondral bone layer is depicted in navy, and superficial 2 mm layer in orange. **(A)** Associations at the palmar MCIII site. **(B)** Associations at the dorsal MCIII site. **(C)** Associations at the proximal sesamoid site.

| 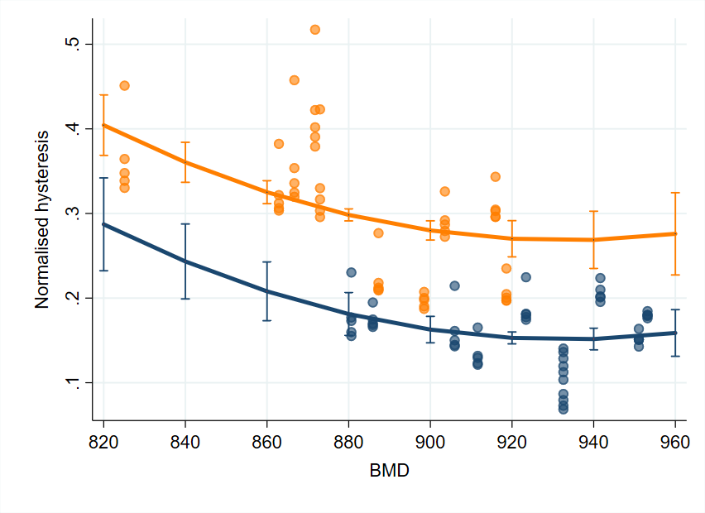   1. Palmar MCIII | 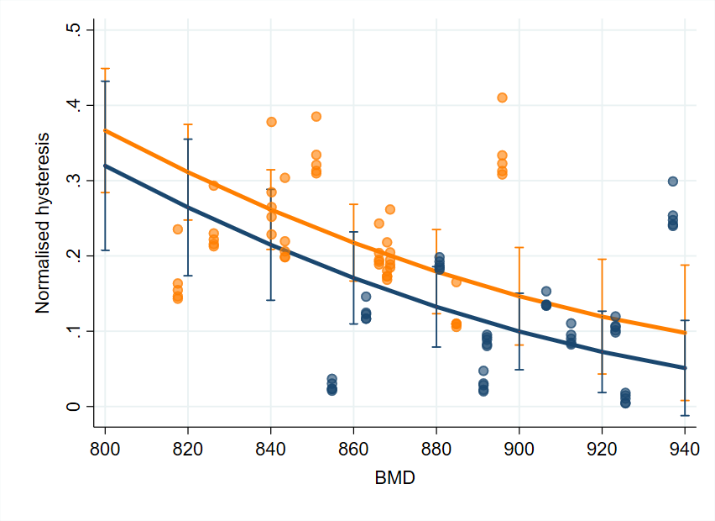   1. Dorsal MCIII |
| --- | --- |
| 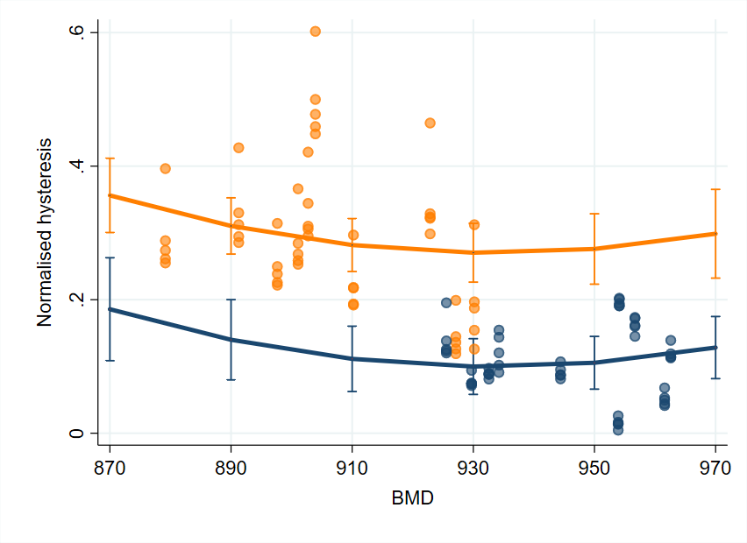   1. Sesamoid |  |

**Figure 4.3.** Adjusted margins plots of the relationship between normalized hysteresis (fraction of energy loss) and bone mineral density (BMD, mg HA/ccm) of subchondral bone in Thoroughbred racehorses (n = 10) with 95% confidence intervals. The deeper 2 mm subchondral bone layer is depicted in navy, and superficial 2 mm layer in orange. **(A)** Associations at the palmar MCIII site. **(B)** Associations at the dorsal MCIII site. **(C)** Associations at the proximal sesamoid site.
